# Developmental differences in social information use under uncertainty: A neurocomputational approach

**DOI:** 10.1016/j.dcn.2025.101604

**Published:** 2025-08-07

**Authors:** Lieke Hofmans, Wouter van den Bos

**Affiliations:** a Department of Developmental Psychology, University of Amsterdam, Amsterdam, the Netherlands; b Motivation, Brain and Behaviour Lab, Paris Brain Institute (ICM), Hôpital de la Pitié-Salpêtrière, Paris, France

**Keywords:** Development, Adolescence, Social learning, Uncertainty, Bayesian decision-making, Neuroimaging, Metacognition

## Abstract

Adolescence is a period of social re-orientation, with studies suggesting that adolescents may be more sensitive to peer influence than other age groups. A clearer understanding of the dynamics and development of peer influence during adolescence is therefore particularly pertinent. In this study, we compared the cognitive and neural processes underlying social learning in adolescents (12–18 years) and adults (22–45 years), focusing on how uncertainty influences social information use. Participants completed a perceptual decision-making task in which they could revise their initial estimate after viewing a peer's estimate. Uncertainty was manipulated by varying the amount of information provided before their decision and by manipulating the peer's reported confidence. Using a combination of model-free analyses and a Bayesian computational model, we found that while adolescents and adults exhibit similar core decision-making mechanisms, computational modeling revealed that adolescents were less sensitive to variations in their own certainty and peer confidence, reducing the effect on social information use. Functional MRI revealed that adolescents showed a reduced neural response to peer confidence variations compared to adults, but exhibited a stronger initial neural response to variations in their own certainty. However, this heightened response was not present anymore when personal and peer information was to be combined. We discuss how these observations might be explained by ongoing neural development during adolescence, leading to reduced metacognitive abilities which hinder the effective integration of precision signals. Together, these findings deepen our understanding of how adolescents process social information under uncertainty and how this process evolves with age.

## 1 Introduction

Adolescents are generally more preoccupied with their peers and more prone to peer influence than in other periods of life (Andrews et al., 2021, Blakemore and Mills, 2014, Somerville, 2013). Peer influence, or social information use, can be very advantageous, such that adolescents can use their peers as sources of information on how to behave adaptively and morally within their changing environment (Bingham et al., 2016, Duell and Steinberg, 2019, Telzer et al., 2018, Molleman et al., 2022). Moreover, imitating others’ behavior can speed up learning, by not having to go through a costly process of trial-and-error (Morin et al., 2021).

However, not all peer information is equally valuable and in not every situation one should be distracted by peers’ opinions. Learning when to rely on peers is a crucial developmental goal for adolescents. The value of using social information is dependent on 1) our current knowledge, and 2) the expertise of the source. First, when we find ourselves in new or uncertain environments, and thus have little knowledge about what to do, we are more inclined to rely on others’ behavior to inform our own actions (Ciranka and van den Bos, 2020; Laland, 2004). In this respect, it might not be very surprising that adolescents are generally more prone to peer information, as they find themselves in a very volatile period in which they encounter many new and unstable (social) situations (Ciranka and van den Bos, 2021, Parr et al., 2024). Indeed, previous empirical studies have found more information search and social information use for adolescents compared to adults (Moutoussis et al., 2016, Niebaum et al., 2022). However, although both adults and adolescents (Slagter et al., 2023) use more social information when they are more uncertain, it is not yet clear how adolescents’ sensitivity to uncertainty compares to that of adults.

Apart from your own uncertainty, the expertise of the other poses an important factor in determining how to weight their information relative to your own. In daily life, however, objective measures of others’ expertise are often lacking. Instead, others often communicate how confident they are, with both children and adults being more likely to use social information from more confident peers (Gradassi et al., 2022a, Wood et al., 2013, Zarnoth and Sniezek, 1997). Although adolescents are generally more focused on their peers (Andrews et al., 2021, Blakemore and Mills, 2014, Somerville, 2013), and they do use social information depending on the peer’s intelligence and social status (da Silva Pinho et al., 2021; Gradassi et al., 2022b; Helms et al., 2014; Wilks et al., 2015), their response to peer confidence has not yet been fully explored.

**Fig. 1:**
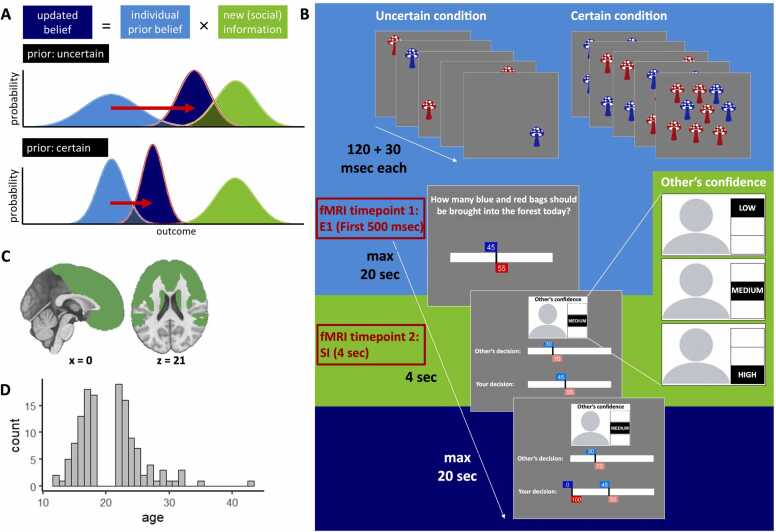
**Bayesian updating under uncertainty**. **A.** An agent combines their prior personal belief or information (light blue) with new social information (green) to arrive at an updated belief (dark blue). Higher uncertainty is represented as a wider distribution. In the case of high prior uncertainty (top panel), the prior information is assigned a relatively low weight, resulting in a stronger impact of the social information (red arrow). Under certainty (bottom panel), the prior information is assigned a relatively high weight, resulting in a less pronounced impact of the same social information. Similarly, if the social information comes from a peer who reports to be very confident, the social information will be assigned a higher weight than when the peer reports not to be confident, resulting in a higher impact of the social information. **B**. Task schematic: Participants first view 5 fields of either 1 (uncertain condition) or 9 (certain condition) mushrooms each, based on which they can submit their first estimate using a slider. This constitutes their individual prior belief (light blue background). Durations of events are indicated next to the timeline. The first 500 ms that the participant is prompted to submit their first estimate is our first timepoint for fMRI modeling. They then see the peer’s estimate and associated confidence report, which can be either low, medium or high. This constitutes the social information (green background), which is our second timepoint for fMRI modeling. Based on the social information, they are then prompted to submit their second estimate, which constitutes their belief update (dark blue background) **C.** The brain mask used for the fMRI analyses included the frontal lobes, insula, TPJ and striatum. **D.** Distribution of age across our experiment.

Previous research strongly suggests social information use can be described as akin to Bayesian updating, where people engage in precision weighting of the various pieces of information (Behrens et al., 2008, Ciranka and van den Bos, 2020, Perreault et al., 2012, Toelch et al., 2014). This means that more certain or reliable information is considered to be more precise and therefore given greater consideration. Thus, when you are more certain about your personal information – that is, outcomes that you have experienced yourself – you will more strongly weight this information compared to social information (Fig. 1A). Conversely, when a peer reports to be very confident, you will translate this into a high precision weight and a stronger belief update based on their information. Apart from Bayesian principles, adolesecents and adults also apply simple heuristics to use social information, including copying peers if they constitute a majority or when they display consistent behavior (Kendal et al., 2018; Molleman et al., 2020; Slagter et al., 2023). For example, when adult participants played a task in which they could update their initial response based on social information, their behavior was best described by a model that included both Bayesian updating to combine personal and social information and a simple stay heuristic to stick with their initial response, without considering the peer’s response (Molleman et al., 2020). It remains unclear how Bayesian processes and heuristic strategies evolve throughout adolescence. While complex reasoning abilities tend to improve with age during this period (Huizenga et al., 2007), there is also evidence that mid-adolescents can exhibit more optimal Bayesian behavior in tasks requiring adaptation to changing environments (Eckstein et al., 2022). Similarly, some heuristics, such as a bias toward random exploration, appear to decrease with age (Dubois et al., 2022), though the direction of change may vary depending on the specific bias in question (Strough et al., 2011).

These developmental shifts in decision-making may be associated with ongoing neural changes. For example, in adults, uncertainty (or precision) has been shown to be represented across a wide network within the brain. Bayesian probability distributions can already be decoded from the sensory cortex (Geurts et al., 2022, van Bergen et al., 2015) Higher cognitive areas, such as the medial prefrontal cortex (mPFC) (Bach and Dolan, 2012, De Martino et al., 2013, Gherman and Philiastides, 2018, Lebreton et al., 2015, Rouault et al., 2023), the anterior cingulate cortex (ACC) (Bang and Fleming, 2018, Fleming et al., 2012), the anterior insula (Bach and Dolan, 2012, Singer et al., 2009) and the ventral striatum (Bang and Fleming, 2018, Hebart et al., 2016) have been implicated in subjective confidence, and the dorsolateral prefrontal cortex (dlPFC) has been implicated in outcome uncertainty and metacognition about uncertainty (Alexander et al., 2023, De Martino et al., 2013, Fleming et al., 2012, Rouault et al., 2023). Many of these areas, including the striatum, mPFC and dlPFC have also been identified in adults in reasoning about the value and uncertainty of others’ information, including peer confidence reports (Burke et al., 2010, Heyes, 2012, Ruff and Fehr, 2014). Other areas, including the temporoparietal junction (TPJ) (Campbell-Meiklejohn et al., 2017, McKell Carter et al., 2012, Saxe and Kanwisher, 2003, Zaki et al., 2016) and the superior temporal sulcus (STS) (Isik et al., 2017, Rushworth et al., 2013) are more specifically involved in social cognition and Theory of Mind (ToM), which involves reasoning about others' intentions and beliefs. For example, the TPJ has been shown to play a role in the tracking of social information (Zhang and Gläscher, 2020) and expertise (Boorman et al., 2013). Moreover, correlates of the integration of personal and social information have been observed in the ventromedial prefrontal cortex (vmPFC) and ventral anterior cingulate cortex (Behrens et al., 2008, Lockwood and Wittmann, 2018, Zhang and Gläscher, 2020).

Functional changes within these neural networks during adolescence can lead to differential processing of uncertainty and peer-related signals between adolescents and adults. Ongoing maturation of the PFC during adolescence (Casey, 2015, Casey et al., 2008, Gogtay et al., 2004) might result in poorer precision-estimates and less accurate forms of metacognition. The question then arises to what extent adolescents’ higher order decision-making resembles Bayesian updating. Not perfectly integrating precision-estimates into their decisions could lead adolescents to be more uncertain in general (Reiter et al., 2021) and to be less sensitive to varying levels of state uncertainty and peer confidence, rendering them more susceptible to peer influence and to using simple decision heuristics instead. The ongoing development of ToM and the TPJ during adolescence (Güroǧlu et al., 2011, Klapwijk et al., 2013, Mills et al., 2014), could additionally contribute to adolescents being less sensitive to peer-related cues. On the other hand, adolescents might be more sensitive to peer cues due to their strong focus on peers, which could be influenced by heightened activation of striatal regions (Blakemore and Robbins, 2012, Crone and Dahl, 2012, Somerville et al., 2011).

Here, we will compare if and how processes related to social information use under uncertainty differ between adolescents and adults. We will use a social information use task, in which participants have the opportunity to update an initial response based on a peer’s response. We vary both the amount of their personal information, affecting their own certainty, as well as the peer’s confidence rating. Based on previous research suggesting that social information use is subject to Bayesian information processing, we compare different Bayesian models to computationally describe participants’ cognitive processes in terms of a precision-modulation of both personal and social information, which is then translated into a Bayesian update. The Bayesian model is then further expanded to include a decision heuristic that captures participants' tendency to stay with their initial response, regardless of the peer's input. Our primary, hypothesis-driven predictions are that adolescents show higher overall social information use and reduced sensitivity to their own certainty. Our predictions for sensitivity to peer confidence are less clear: adolescents may be either less sensitive due to immature (meta)cognition or more sensitive due to heightened peer focus. We also explore neural activity during the task using fMRI to investigate how neural responses to personal and peer-related certainty differ between adolescents and adults. Given the previously discussed neural differences in decision-making between these age groups, we are particularly interested in examining activity within the PFC, insula, TPJ, and striatum.

## 2 Methods

### 2.1 Participants

One hundred four adult (22–45 years old) and 105 adolescent (12–18 years old) participants were recruited via the research participant system of the University of Amsterdam, Instagram or posters at schools in the vicinity of the research center. Participants were right-handed, Dutch-speaking, screened for MR compatibility, had normal or corrected-to-normal vision and had completed or were following a university-level education (adults) or had completed or were following pre-university-level education (adolescents). The experiment described in the current study was part of a larger task battery, consisting of a session from home (30 min) during which participants filled in a questionnaire about their digital device and social media use and a behavioral-fMRI session at the research center (2.5 h) during which they completed a social information use task in the MR scanner (described below) as well as a spatial working memory task, a reversal learning task, an information search task and a shorter social information use task. This latter task, the Berlin Estimate Adjustment Task (Molleman et al., 2019, 2020), was performed approximately 30 min after completing the task in the scanner. Similar to the task described in the current manuscript, participants estimated the number of animals shown on screen, viewed the estimate of an unknown peer, and had the opportunity to revise their response. Participants were paid 40 euro plus a bonus contingent on their performance, ranging between 0.70 and 2.90 euro for all tasks combined, and ranging between 0 and 1.50 for the fMRI task. All participants performed the social information use task inside the MR scanner, and both behavioral and fMRI analyses were conducted on data from this single task. Out of the participants who signed up for the study, a total of 74 adults and 70 adolescents completed their fMRI session. From the adult group, 2 were excluded due to incomplete datasets, one was excluded because they fell outside our predefined age range, 3 participants were excluded based on our behavioral exclusion criteria described below, and a further 10 were excluded from the fMRI analysis because they did not pass our quality assessment (9) or because their timing files were unavailable (1), resulting in 68 participants included in the behavioral analysis (27 men, 40 women, and 1 participant who identified as non-binary; age: mean (SD) = 24.8 (3.7); Fig. 1D) and 58 participants included in the fMRI analysis (22 men, 35 women, and 1 participant who identified as non-binary; age: mean (SD) = 24.3 (3.5)). From the adolescent group, 6 participants were excluded based on our behavioral exclusion criteria and a further 17 were excluded from the fMRI analysis because they did not pass our quality assessment, resulting in 64 participants included in the behavioral analysis (23 men, 39 women, and 2 participant who identified as non-binary; age: mean (SD) = 16.4 (1.5)) and 47 participants included in the fMRI analysis (18 men, 27 women, and 2 participant who identified as non-binary; age: mean (SD) = 16.4 (1.5); Fig. 1D). The procedure of both studies was approved by the local ethics committee (Adults: 2021-DP-13756; Adolescents: 2021-DP-13738). Before the start of the experiment, participants, and their parents in case the participant was younger than 16 years old, gave written consent according to the declaration of Helsinki.

### 2.2 Social information use task

The social information use task is an estimation game modelled after the marble task (Phillips and Edwards, 1966) and the Berlin Estimate AdjuStment Task (BEAST; (Molleman et al., 2019)) and probes the amount of social information use as a function of own and others decision confidence. The game was programmed in Python 3.6.6 using PsychoPy. A schematic of the game and corresponding timings is schematically depicted in Fig. 1B.

Participants were instructed that they are part of a community for which the main food resource is mushrooms. These mushrooms are harvested from a forest in the vicinity of their village. Soon, it will be time to harvest, and the leader of the community has asked them for their help in coordinating this. There are two types of mushrooms, blue and red ones. Their task at the start of each day (trial) is to estimate the percentage of blue mushrooms in the forest. To make an informed guess, they will walk through 5 fields that surround their village. The mushrooms in the fields are a good, but not exact representation of what will be found in the forest on that day. On some days they encounter many mushrooms (9 mushrooms per field, 45 in total) in the fields and on other days they encounter fewer mushrooms (1 mushrooms per field, 5 in total). The more mushrooms they find in the nearby fields, the more information they have to base their estimate on and the more certain they can be. Participants submitted their initial estimate using a 100-point slider (ranging from 0 to 100) operated via an MR-compatible button box. This estimate reflected their belief about the proportion of blue mushrooms, with the red mushroom value automatically set so the total equaled 100. After submitting this initial estimate, participants were shown peer information stemming from a peer described as a previous participant in the study. No additional information about the peer was provided. The percentage of blue and red mushrooms in the forest was the same for this other person, but they were from a different village on the other side of the forest, meaning that they had to sample from different fields on the other side of the forest, and the number of blue and red mushrooms in these fields might have been different from those in the fields surrounding the participants village. The participant was first shown the peer’s confidence rating in their estimate, which could be either low, medium or high. After 4 s, this information was accompanied by the peer’s estimate. Based on this new information, the participant could adapt their estimate. The peer’s estimate and confidence rating stemmed from real previous participants, who had played a version of the mushroom game without any social information. The peer estimate was chosen such that, if possible, it was in the direction of the true underlying percentage and the difference between the participants first estimate and the peer estimate ranged between 15 and 18 percentage points. This range was selected to leave enough room for adjustment of the estimate and control for distance that might influence the amount of adjustment (Gradassi et al., 2022a, Gradassi et al., 2022b, Molleman et al., 2019, Molleman et al., 2020, da Silva Pinho et al., 2021).

Participants completed 75 trials, pseudo-randomly divided across 3 runs, which lasted approximately 30 min in total. Sixty trials were equally divided across own certainty (certain, uncertain), peer confidence (low, medium, high) and true percentage of blue versus red mushrooms (12.5, 25, 37.5, 62.5, 75). Another set of 15 “filler trials” was included to make the trials more realistic: the true percentage could range from 0 to 100 and the peer’s estimate was either very close (< 3) or very far (> 40) from the participant’s first estimate. The maximum time to submit an estimate was 20 s, after which the trial would time out and the participant would see a message stating that they were too late. No feedback was given on their estimates, but participants could earn a bonus depending on their performance. One estimate of one trial was randomly selected. If this estimate was exactly the same as the true percentage, they would earn 1.50 pounds, of which 0.04 pounds was subtracted for each percentage point off. Their bonus could never go below 0.

The main task was preceded by instructions, 2 short rounds of 3 practice trials each, and a longer practice round of 15 trials outside the scanner. During this practice round, participants were asked to rate their own confidence after submitting their first estimate as either low, medium or high, to give them a better understanding of what the peers confidence ratings entailed. Moreover, a quick analysis of this practice round informed the experimenter about whether the participant indeed understood the game before going into the scanner. Specifically, if a participant’s initial estimates followed the absolute number of blue mushrooms rather than the ratio of blue to red mushrooms, or if their estimates showed no sensitivity to the ratio, the task was explained again. The participant was then asked to explain the task back to the experimenter to ensure they had understood it correctly.

### 2.3 MRI acquisition and preprocessing

The fMRI experiment was performed on a Philips Achieva 3 T MRI scanner and a 32-channel SENSE headcoil at the imaging center at the Roeterseiland campus of the University of Amsterdam. First, a 6-minute whole-brain high-resolution structural image was acquired for anatomical referencing, using a T1-weighted magnetization prepared, rapid-acquisition gradient echo (MPRAGE) sequence (TR = 8.2 ms, TE = 3.7 ms, flip angle = 8°, 220 axial slices in ascending order, field of view = 240 × 188 mm, voxel size = 1 × 1 × 1 mm). Images with blood-oxygen level-dependent (BOLD) contrast were acquired in 3 runs, using a whole-brain T2*-weighted single-shot gradient-echo multi-echo echo planar imaging (EPI) sequence (TR = 2000 ms, TE = 28 ms, flip angle = 76.1°, 36 axial slices per volume in ascending order, field of view = 240 × 240, reconstruction matrix = 80 × 80, voxel size = 3 × 3 × 3 mm, slice gap = 0.3 mm). After each functional run, a phase-encoding polarity (“topup”) gradient-echo sequence consisting of 3 volumes was acquired to estimate the static magnetic field’s inhomogeneity. The parameters were exactly the same as for the corresponding functional scans, but with a reversed phase-encoding direction.

All images were converted from PAR/REC to NIfTI format and into BIDS (Brain Imaging Data Structure) (Gorgolewski et al., 2016) using BIDScoin (Zwiers et al., 2022). The images were preprocessed using HALFpipe (Harmonized AnaLysis of Functional MRI pipeline) version 1.2.2 (Waller et al., 2022), which is based on fMRIPrep (Esteban et al., 2019). Structural images were corrected for bias field, skullstripped and segmented before being registered to the MNI152NLin2009cAsym standard template. Preprocessing of the functional images included realignment to the middle volume, slice timing correction, susceptibility distortion correction based on the topup scans, coregistration to the participant’s structural scan and spatial normalization to MNI152NLin2009cAsym space. Denoising was then performed by spatial smoothing using a 6 mm FWHM kernel, grand mean intensity normalization with a mean of 10 000 and temporal filtering using a high pass filter width of 100 s. Physiological nuisance regressors were extracted using aCompCor (Behzadi et al., 2007). See https://fmriprep.readthedocs.io/en/0.6.2/workflows.html for more details on the preprocessing pipeline. Exclusion after quality assessment was based on a visual check of the skull stripping, spatial normalization, EPI tSNR, EPI confound and head motion results (excessive motion was defined as mean FD > 0.5 mm or max FD > 3 mm).

### 2.4 Behavioral data analysis

Our main outcome measure was social information use (s), defined as the adjustment from the first (E_1_) to the second (E_2_) estimate, relative to the peer estimate (P):

(1) $$s=\frac{E_{2}-E_{1}}{P-E_{1}}$$

A value of 0 indicates no social information use, 1 indicates complete copying of the peer, negative values reflect adjustment away from the peer, and values above 1 indicate over-adjustment beyond the peer estimate. Participants who did not understand the task, as assessed per qualitative visual inspection, were excluded from the analysis (e.g. participants who responded randomly to differences in percentage of blue versus red mushrooms, or participants who did not extrapolate the number of seen mushrooms to a total of 100), as were participants whose social information use was 0 at more than 70 % of the trials, as this would yield minimal variation to conduct the analyses. Filler trials, missed trials (without a response at either the first or second estimation phase), trials in which no suitable social information (i.e. within the correct range from the participant’s first estimate) could be sampled from our database, trials for which either the first or the second estimate deviated more than three standard deviations from the group mean, grouped per own certainty condition, ratio and age group, and trials in which social information use did not range between 0 and 1, meaning that it was not a weighted average of the participant’s first estimate and the peer’s estimate,^1^ were excluded from the analysis (Table S1).

The behavioral analyses were conducted in R version 4.0.4 (R Core Team, 2021). To assess the combined effects of own certainty and peer confidence on social information use, we conducted a linear mixed effects regression, using the lmer function from the *lmerTest* package (Kuznetsova et al., 2017). Own certainty and peer confidence (with mean-centered values −1 for low, 0 for medium and 1 for high peer confidence), as well as their interaction, were included as both fixed and random effects:

$$s∼\mathrm{own certainty}\left(\mathrm{ref}=\mathrm{uncertain}\right)*\mathrm{peer confidence}+\left(1+\mathrm{own certainty}*\mathrm{peer confidence}\right|\mathrm{participant})\;$$

To compare age groups, we ran the same analysis again while including the categorical variable group (adolescents or adults) as a fixed effect, both as a main effect and in interaction with the other predictors:

$$s∼\mathrm{own certainty}\left(\mathrm{ref}=\mathrm{uncertain}\right)*\mathrm{peer confidence}*\mathrm{group}\left(\mathrm{ref}=\mathrm{adults}\right)+\left(1+\mathrm{own certainty}*\mathrm{peer confidence}\right|\mathrm{participant})$$

Intraclass correlation coefficients (ICCs) were computed using the *performance* package (Lüdecke et al., 2021) to quantify the proportion of variance attributable to between-subject differences, and reported marginal and conditional R² to quantify the variance explained by the fixed effects alone and by the full model, respectively. Standardized beta coefficients (β) were computed using the standardize_parameters() function from the *parameters* package (Lüdecke et al., 2020).

### 2.5 Control analyses

To verify that any group differences in social information use could not be attributed to differences in performance, we ran a linear mixed effects regression on performance. Performance was indexed by deviance, defined as the absolute difference between the participant’s first estimate and the true percentage of blue mushrooms. The model included age group and own certainty as fixed effects, along with their interaction. To account for within-subject variability, we included random intercepts and random slopes for own certainty at the participant level:

$$\mathrm{deviance}∼\mathrm{own certainty}\left(\mathrm{ref}=\mathrm{uncertain}\right)*\mathrm{group}\left(\mathrm{ref}=\mathrm{adults}\right)+\left(1+\mathrm{own certainty}\right|\mathrm{participant})\;$$

To further explore whether differences in performance, and possibly in participants’ subjective confidence, could underlie age differences in the effect of own certainty, we conducted an additional analysis using confidence ratings collected during the practice phase. Specifically, we tested whether adolescents differ from adults in their ability to align confidence with performance. For each participant, we computed the correlation between deviance and confidence ratings, after which we compared the resulting subject-specific R² values across age groups using a *t*-test.

To test whether potential age group differences in residual variance affected our results, we conducted a control analysis using the lme function from the *nlme* package (Pinheiro et al., 2025), allowing for different residual variances by age group. See Supplementary Section *Modeling Heteroscedasticity* for more details.

In the above analyses, we treated age as a categorical variable (adolescents vs adults), which we considered the most appropriate approach given the relatively discrete age clusters in our sample (16–18 vs 22–23 years; see Fig. 1D). This group-wise approach allowed for clear hypothesis testing regarding age-related differences in social information use and neural mechanisms. However, to assess the robustness of our findings, we also conducted supplementary analyses using age as a continuous predictor (see Supplementary Section *Age as a continuous predictor*).

### 2.6 Computational modeling

We used a computational modeling approach to quantify Bayesian processes that we hypothesized to underly participants’ choices (Fig. 1A). Apart from a base model M0, we developed nine models with increasing complexity to investigate whether additional parameters explained the observed behavior better. The model-building process was conditional: If a model performed worse compared to the preceding model (based on the Bayesian Information Criterion; see *Model fitting procedure* below), we did not use it as the basis for further model development. Instead, the next model in the sequence was built upon the last accepted model. This iterative approach ensured that each new model only added complexity if it improved the model fit. Parameter bounds were chosen based on pilot data (Gershman, 2016) to avoid edge effects in posterior distributions while allowing a wide range of plausible behaviors (e.g., α and θ parameters could produce subjective mushroom counts well beyond the actual stimuli, up to 4500 for self-estimates in the certain condition and 1500 for peer estimates under high peer confidence).

An overview of all models can be found in Table 1. For all models, we assumed a beta distribution over all possible response options for their E2 (0–100 %, divided by 100). We set an initial prior of 1 blue and 1 red mushroom, to which we then added the participant’s estimate of blue and red mushrooms to arrive at a posterior for E1, where we further assumed that participants’ E1 was an accurate representation of the percentage of blue mushrooms they had seen in the fields:

$${E1}_{\mathrm{blue}}=1+\frac{E1}{100}\times N$$

(2) $$\;{\;E1}_{\mathrm{red}}\;=1+\left(1-\frac{E1}{100}\right)\times N$$

where N is the total number of mushrooms that the participant saw on that trial, being either 5 for the uncertain condition and 45 for the certain condition. For example, if 45 mushrooms were shown in a particular trial and the E1 was 20, meaning that the participant estimated that the percentage of blue mushrooms was 20 %, the number of blue mushrooms was set to 20/100 × 45, which was then added to the prior of 1, resulting in ${E1}_{\mathrm{blue}}=10$. The number of blue and red mushrooms as seen by the peer follow from the peer’s estimate P and the total number of mushrooms seen by the peer. Because the total number of mushrooms seen by the peer is unknown to the participant, we set this to 25 in **M0**, which is the average of the total number seen by the participant, across all trials.

$$P_{\mathrm{blue}}=\frac{P}{100}\times N_{\mathrm{peer}}$$

(3) $$\;P_{\mathrm{red}}=\left(1-\frac{P}{100}\right)\times N_{\mathrm{peer}},\;\;N_{\mathrm{peer}}=25$$

**Table 1:** Computational models.

**Model**	**Description**	**# Par.**	**Parameters**	**ΔBIC**	
				**Adolescents**	**Adults**
M0	Base model	0	-	3968	4693
M1a	M0 + modulation of the weight of E1	1	0.1≤α≤100	3174	3644
M1b	M0 + modulation of the weight of E1, separately for the low and high own certainty condition	2	$\begin{array}{c} 0.1\leq \alpha_{\mathrm{uncertain}}\leq 100\;\\ 0.1\leq \alpha_{\mathrm{certain}}\leq 100 \end{array}$	1906	2940
M2a	M1b + modulation of the weight of the peer estimate	3	$\begin{array}{c} 0.1\leq \alpha_{\mathrm{uncertain}}\leq 100\;\\ 0.1\leq \alpha_{\mathrm{certain}}\leq 100\;\\ 0.1\leq \theta \leq 1000 \end{array}$	1570	2152
M2b	M1b + modulation of the weight of the peer estimate, linearly dependent on peer confidence level	4	$\begin{array}{c} 0.1\leq \alpha_{\mathrm{uncertain}}\leq 100\;\\ 0.1\leq \alpha_{\mathrm{certain}}\leq 100\;\\ 0.1\leq \theta_{\mathrm{IC}}\leq 500\;\\ 0.1\leq \theta_{\mathrm{slope}}\leq 500 \end{array}$	394	143
M2c	M1b + modulation of the weight of the peer estimate, separately for each peer confidence level	5	$\begin{array}{c} 0.1\leq \alpha_{\mathrm{uncertain}}\leq 100\;\\ 0.1\leq \alpha_{\mathrm{certain}}\leq 100\;\\ 0.1\leq \theta_{\mathrm{low}}\leq 1000\;\\ 0.1\leq \theta_{\mathrm{medium}}\leq 1000\;\\ 0.1\leq \theta_{\mathrm{high}}\leq 1000 \end{array}$	725	364
M3a	M2b + overall stay bias	5	$\begin{array}{c} 0.1\leq \alpha_{\mathrm{uncertain}}\leq 100\;\\ 0.1\leq \alpha_{\mathrm{certain}}\leq 100\;\\ 0.1\leq \theta_{\mathrm{IC}}\leq 500\;\\ 0.1\leq \theta_{\mathrm{slope}}\leq 500\;\\ 0.01\leq \beta \leq 0.99 \end{array}$	167	246
M3b	M2b + stay bias, separately for the low and high own certainty condition	6	$\begin{array}{c} 0.1\leq \alpha_{\mathrm{uncertain}}\leq 100\;\\ 0.1\leq \alpha_{\mathrm{certain}}\leq 100\;\\ 0.1\leq \theta_{\mathrm{IC}}\leq 500\;\\ 0.1\leq \theta_{\mathrm{slope}}\leq 500\;\\ 0.01\leq \beta_{\mathrm{uncertain}}\leq 0.99\;\\ 0.01\leq \beta_{\mathrm{certain}}\leq 0.99 \end{array}$	482	632
M3c	M2b + stay bias, linearly dependent on peer confidence level	5	$\begin{array}{c} 0.1\leq \alpha_{\mathrm{uncertain}}\leq 100\;\\ 0.1\leq \alpha_{\mathrm{certain}}\leq 100\;\\ 0.1\leq \theta_{\mathrm{IC}}\leq 500\;\\ 0.1\leq \theta_{\mathrm{slope}}\leq 500\;\\ 0.01\leq \beta \leq 0.99 \end{array}$	21	55
**M3d**	**M2b + stay bias, exponentially dependent on peer confidence level**	**5**	$\begin{array}{c} 0.1\leq \alpha_{\mathrm{uncertain}}\leq 100\;\\ 0.1\leq \alpha_{\mathrm{certain}}\leq 100\;\\ 0.1\leq \theta_{\mathrm{IC}}\leq 500\;\\ 0.1\leq \theta_{\mathrm{slope}}\leq 500\;\\ 0.01\leq \beta \leq 0.99 \end{array}$	**0**	**0**

ΔBIC = Bayesian information criterion relative to the best fitting model (lower BIC value indicates better fit); M3d (in bold) is the best fitting model.

Adding these peer’s $P_{\mathrm{blue}}$ and $P_{\mathrm{red}}$ to the participant’s ${E1}_{\mathrm{blue}}$ and ${E1}_{\mathrm{red}}$, respectively, results in the posterior for E2:

$${E2}_{\mathrm{blue}}={E1}_{\mathrm{blue}}+P_{\mathrm{blue}}$$

(4) $${\;E2}_{\mathrm{red}}={E1}_{\mathrm{red}}+P_{\mathrm{red}}\;$$

One can then define a probability density function (PDF) of the posterior beta distribution based on ${E2}_{\mathrm{blue}}$ and ${E2}_{\mathrm{red}}$, for 0≤x≤1 and shape parameters ${E1}_{\mathrm{blue}}$ and ${E1}_{\mathrm{red}}$:

(5) $$f\left(x;{E2}_{\mathrm{blue}},{E2}_{\mathrm{red}}\right)=\frac{x^{{E2}_{\mathrm{blue}}-1}\times {\left(1-x\right)}^{{E2}_{\mathrm{red}}-1}}{B({E2}_{\mathrm{blue}},{E2}_{\mathrm{red}})}$$

where the beta function B is a normalization constant to ensure that the total probability adds up to 1, which we then used for our model fitting procedure (see more details below).

### 2.7 Model family 1: Individual modulation of the weight of E1

Participants might under- or overestimate the numerosity of displayed items or their own confidence (Krueger, 1984, Lichtenstein and Fischhoff, 1977). In **M1a**, we therefore tested whether adding a parameter α that modulates the perceived number of total mushrooms, effectively modulating the subjective weight of the E1 at the individual level, improved model performance:

(6) $$N_{\mathrm{perceived}}=N\times \alpha$$

In **M1b**, we extended this modulation by including a dual α, which separately modulated the perceived number of total mushrooms for the uncertain and certain conditions:

(7) $$\begin{array}{c} N_{perceived}=\left\{\begin{array}{l} N\times \alpha_{uncertain}\;\;\;if\;uncertain\;condition\\ N\times \alpha_{certain}\;\;\;\;\;\;if\;certain\;condition \end{array}\right)\\ \; \end{array}$$

### 2.8 Model family 2: Individual modulation of the weight of the peer estimate

Next, we tested whether individually modulating the number of mushrooms the participant assumed the peer had seen improved model performance. In **M2a**, rather than fixing the number of mushrooms as seen by the peer to 25, we introduced θ that modulated this number per participant:

(8) $$N_{\mathrm{peer}}=\theta$$

In **M2b**, we tested whether model performance improved when the modulation by θ linearly depended on the confidence level reported by the peer, such that the weight of the peer estimate increased as the peer reported to be more confident:

(9) $$N_{\mathrm{peer}}=\theta_{\mathrm{IC}}+\theta_{\mathrm{slope}}\times {(\mathrm{confidence}}_{\mathrm{peer}}-1)$$

where θ_IC_ is the intercept representing the baseline influence of the peer when confidence is at its lowest level, and θ_slope_ captures how much the influence increases with each step in confidence. Peer confidence level was defined as 1 for low, 2 for medium and 3 for high confidence. In **M2c**, we tested whether a modulation by θ for each peer confidence level separately, allowing for a non-linear modulation, improved model fit:

(10) $$N_{\mathrm{peer}}=\left\{\;\begin{array}{c} \theta_{\mathrm{low}}\;\;\;\;\;if\;peer\;confidence\;is\;low\;\;\;\;\;\;\;\\ \theta_{\mathrm{medium}}\;\;\;if\;peer\;confidence\;is\;medium\\ \theta_{\mathrm{high}}\;\;\;\;\;\;\;if\;peer\;confidence\;is\;high\;\;\;\;\;\;\; \end{array}\;\right)\;\;\;$$

### 2.9 Model family 3: stay bias

A visual inspection of the data made clear that participants exhibited a tendency to stay with their first estimate (s = 0), a response pattern that stood apart from the rest of their near-normally distributed response pattern. This suggests that at least at some trials, participants exhibited a bias in staying with their first estimate, without even processing the peer’s estimate, in line with earlier findings suggesting that people combine Bayesian processing with simple heuristics. To allow for this bias, we added a stay bias to models M3. For **M3a** the stay bias, defined by parameter β, was non-selective, such that it was set per individual, but equal across all trials within-participant. **M3b** included a separate parameter β for each own certainty condition, $\beta_{\mathrm{uncertain}}$ and $\beta_{\mathrm{certain}}$. For **M3c** and **M3d**, the stay bias depended on peer confidence level in a linear or exponential fashion, respectively:

(11) $$\mathrm{stay bias}=\left\{\;\begin{array}{c} \beta \;\\ \beta_{\mathrm{uncertain}}\mathrm{or}\beta_{\mathrm{certain}}\\ \beta /confidence_{peer\;}\\ \beta^{confidence_{peer}}\; \end{array}\right)\begin{array}{c} \mathrm{for}M3a\\ \mathrm{for}M3b\;\\ \mathrm{for}M3c\\ \mathrm{for}M3d \end{array}\;$$

For these M3 models, the PDF was then multiplied by (1−stay bias), such that the sum of the stay bias and the total area under the curve (the probability) of the PDF adds to 1. The probability of a stay response at E2 was then estimated by the sum of the stay bias and the probability of s = 0 under the PDF. This means that a stay response could be either the result of a simple stay bias, or of a decision process that considered the peer’s estimate. On non-stay trials, the probability of participants’ responses was estimated solely by the probability of the E2 response under the PDF (multiplied by 1−stay bias).

To summarize, this last model combines Bayesian integration of personal and social information with a stay bias component. Participant’s own evidence is transformed into subjective mushroom counts:

$${E1}_{\mathrm{blue}}=1+\frac{E1}{100}\times N\times \alpha_{\mathrm{own certainty}},\;$$

$${E1}_{\mathrm{red}}=1+\left(1-\frac{E1}{100}\right)\times N\times \alpha_{\mathrm{own certainty}}$$

where E1 is the participant’s first estimate (0−100), N is the total number of mushrooms shown and α modulates certainty differentially for each of the two own certainty conditions.

Next, the peer’s estimate P is transformed into subjective mushroom counts linearly based on the peer’s confidence rating using a θ intercept and slope:

$$P_{\mathrm{blue}}=\frac{P}{100}\times \left(\theta_{\mathrm{IC}}+\theta_{\mathrm{slope}}\times \left(\mathrm{confidence peer}-1\right)\right),$$

$$P_{\mathrm{red}}=\left(1-\frac{P}{100}\right)\times \;\left(\theta_{\mathrm{IC}}+\theta_{\mathrm{slope}}\times \left(\mathrm{confidence peer}-1\right)\right)$$

The posterior belief is then computed as a Beta distribution, which defines a probability density over all possible values of social information use ranging from 0 (fully relying on one's own estimate) to 1 (fully copying the peer). with shape parameters:

$${E2}_{\mathrm{blue}}=\;{E1}_{\mathrm{blue}}+P_{\mathrm{blue}},\;{E2}_{\mathrm{red}}=\;{E1}_{\mathrm{red}}+P_{\mathrm{red}}$$

Finally, the model includes a stay bias β to account for ignoring the peer information entirely. This results in a mixture distribution that combines a Beta distribution with a delta function at zero social information use:

$$\left(1-\beta^{\mathrm{confidence peer}}\right)\times \mathrm{Beta}\left({E2}_{\mathrm{blue}},\;{E2}_{\mathrm{red}}\right)+\beta^{\mathrm{confidence peer}}\times \delta (0)$$

### 2.10 Model fitting procedure

Parameter estimations were performed by individually fitting the model predictions to each participant’s behavior, specifically their E2 responses. The fitting procedure relied on a combination of grid search and maximum likelihood estimation using the L-BFGS-B algorithm implemented in the optim function in R. The log likelihood of the combination of parameter values corresponding to each point on the grid was estimated and minimized. Model comparison was evaluated by computing the Bayesian Information Criterion (BIC), which penalizes for increasing model complexity, across all participants for each of the models. Lower BIC values indicate a better fit. To evaluate differences in cognitive processes between adolescents and adults, we subsequently tested each parameter for statistical differences between age groups using Mann-Whitney *U* test. In order to account for multiple comparisons, we applied the Benjamini-Hochberg (BH) correction to control the false discovery rate.

As for our model-free analyses, we also conducted supplementary analyses using age as a continuous predictor (see Supplementary Section *Age as a continuous predictor*).

### 2.11 Model recovery and parameter recovery

For quality control, we ran a model recovery procedure using nine models *(excluding base model M0).* For each model, we simulated a dataset consisting of 132 simulated participants (68 corresponding to the Adult group and 64 corresponding to the Adolescent group). To simulate realistic parameter values, we sampled from a uniform distribution bounded by the minimum and maximum values observed in the real data after excluding outliers beyond ±3 standard deviations. Each of these simulated datasets were then fit by all nine models – resulting in 84 model fitting procedures. These fits were compared regarding their goodness-of-fit (BIC) and a confusion matrix was constructed to quantify model recoverability (Wilson and Collins, 2019).

We further ran a parameter recovery procedure on all our models, where we assessed the correlation between the ‘true’ parameter estimates, which we defined as the simulated parameter values, and the fitted parameter estimates, which were generated by rerunning each model on simulated data based on these ‘true’ estimates in combination with the same settings and trials as in the experiment.

### 2.12 fMRI data analysis

FMRI data processing was carried out using FEAT (FMRI Expert Analysis Tool) Version 6.00, part of FSL (FMRIB's Software Library, www.fmrib.ox.ac.uk/fsl). Time-series statistical analysis was carried out using FILM with local autocorrelation correction (Woolrich et al., 2001). For each run of each subject, BOLD responses were modeled using event-related GLMs. The first GLM included two parametric regressors: one that modeled model-based own certainty at the time of the E1 (i.e. the prior; first 500 ms, so brain activity would reflect (un)certainty rather than motor activity; Fig. 1B) and one that modeled model-based peer confidence at the time of the social information (duration of 4000 ms). Model-based own certainty was the actual number of mushrooms that was shown on that trial multiplied by either $\alpha_{\mathrm{uncertain}}$ or $\alpha_{\mathrm{certain}}$ (see Eq. 7), depending on the trial condition. Model-based peer confidence was defined as $\theta_{\mathrm{IC}}+\theta_{\mathrm{slope}}\times {(\mathrm{confidence}}_{\mathrm{peer}}-1),\;$with peer confidence set as low = 1, medium = 2, high = 3 (see Eq. 9). We reasoned that model-based own certainty and peer confidence, based on the parameter values resulting from our best fitting Bayesian model, yielded more individualized results. However, we also ran a supplemental GLM that included two regressors relating to the model-free conditions of own certainty, modeled at the time of the E1 (first 500 ms), one for the certain and one for the uncertain condition, and one regressor relating to model-free peer confidence modeled at the time of the social information (duration of 4000 ms). The latter was parametrically modulated by the peer confidence level. We created contrasts for the effect of own certainty (certain – uncertain at E1) and the effect of peer confidence (parametric effect at social information).

The parametric regressors were mean-centered across all trials within a run. Of note, peer confidence, which was modeled at the time of social information, only included non-filler trials, because filler trials contained peer information (very close or very far from the E1) that might have elicited different cognitive processes. In both models, we additionally included temporal derivatives, six motion parameters, framewise displacement, global white matter and CSF signal and the first 6 anatomical principal component noise regressors (aCompCor) as regressors of no interest. All regressors were convolved with a double-gamma hemodynamic response function.

The resulting contrast images per run were averaged at the participant level, which was carried out using a fixed effects model, by forcing the random effects variance to zero in FLAME (FMRIB's Local Analysis of Mixed Effects) (Beckmann et al., 2003, Woolrich, 2008, Woolrich et al., 2004). The participant-level contrasts were subsequently taken to the group level, carried out using FLAME stage 1 to account for both within- and between subject variability. We first included all participants in a combined analysis to identify brain regions activated across the entire sample using a one-sample *t*-test. We then performed a direct comparison between adolescents and adults using two-sample t-tests to identify clusters showing significant differences in brain activation between the two groups. To further explore the clusters identified in the group comparison, we conducted separate follow-up analyses for adolescents and adults, each using a one-sample *t*-test. This allowed us to determine whether the clusters identified in the group comparison exhibited significant activation within each group individually. Normalized (Z-scored) statistic images were thresholded using clusters determined by Z > 3.1 and a (corrected) cluster significance threshold of P = 0.05 (Worsley, 2001). For the group-level analyses, we applied a mask that included the frontal lobe and insula, derived using the MNI structural atlas in FSLeyes, as well as the TPJ and the striatum, based on connectivity-analyses (Mars et al., 2012, Piray et al., 2017) (Fig. 1C). Using these predefined regions provided practical and standardized anatomical boundaries while ensuring complete coverage of the areas of interest described above. However, to also give a more complete overview, results pertaining to age differences at the level of the whole-brain can be found in the supplemental material (Table S8-9).

## 3 Results

### 3.1 Effect of own certainty and peer confidence on social information use

**Fig. 2:**
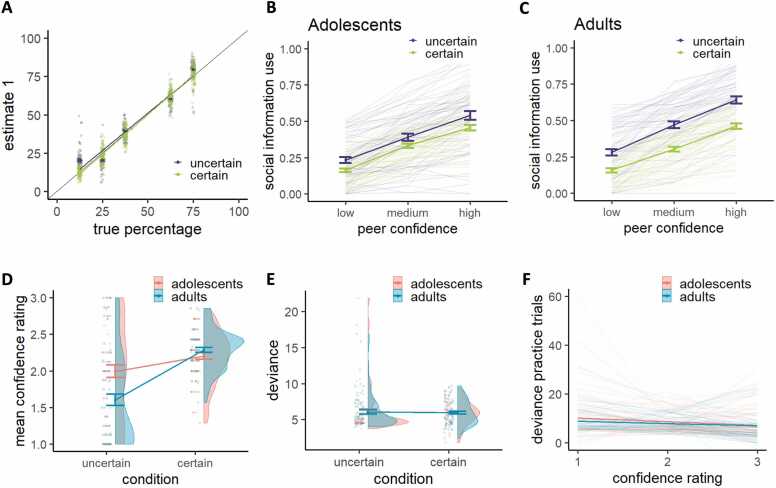
**Effects of own certainty and peer confidence on social information use**. **A.** A depiction of the true percentage of blue mushrooms (x-axis) versus participants’ first estimate (y-axis) shows that participants were fairly accurate. Each individual datapoint represents a participant, whose measure is averaged for each condition (uncertain and certain) and each true percentage separately. **B.** Social information use per own certainty and peer confidence level for adolescents. Thin lines represent individual participants. **C.** Social information use per own certainty and peer confidence level for adults. Thin lines represent individual participants. **D.** Mean self-reported confidence rating for adolescents and adults, averaged per own certainty condition, collected during the 15 practice trials. **E.** Performance, indexed as the absolute deviance of the first estimate from the true percentage of blue mushrooms, as a function of own certainty and age group. **F.** Performance (deviance) on the practice trials as a function of participants’ confidence rating. Error bars represent the standard error around the mean across participants within each group.

For both groups, participants first estimates (E1) clearly followed the true percentages, indicating that they understood the game (Fig. 2A). The average social information use across all trials was 0.37 (Fig. 2B,C), which is comparable to studies using similar designs (Gradassi et al., 2022a, Molleman et al., 2019, Yaniv, 2004). This is consistent with an egocentric bias, where participants place more weight on their personal information than on the peer’s: a one-sample *t*-test confirmed that average social information use was significantly lower than 0.5 (*t*(131) = -11.52, *p* < 0.001). Our linear mixed effect regression model yielded an adjusted intraclass correlation coefficient (ICC) of 0.505, indicating that approximately 50 % of the variance was attributable to between-subject differences. The fixed effects alone explained 29 % of the variance (marginal R^2^ = 0.286), while the combined fixed and random effects explained 65 % of the variance (conditional R^2^ = 0.647). Across groups, social information use was higher in the uncertain compared to the certain condition (*b* = −0.12, β = −0.22, *p* < 0.001; Fig. 2B,C) and higher with increasing peer confidence (*b* = 0.16, β = 0.49, *p* < 0.001). There was also an interaction between own certainty and peer confidence (*b* = −0.02, β = −0.03, *p* = 0.009), such that the effect of peer confidence was stronger under uncertainty compared to certainty. The data thus indicate that participants displayed an egocentric bias, where they assigned roughly twice as much importance to their personal information compared to the peer's information. Only when they were uncertain themselves and the peer reported to be highly confident, they assigned equal or more weight to the peer information than to their personal information.

### 3.2 Age-related differences in sensitivity to own certainty and peer confidence

Our linear mixed effect regression model including age group as a regressor yielded an adjusted intraclass correlation coefficient (ICC) of 0.499, indicating that approximately 50 % of the variance was attributable to between-subject differences. The fixed effects alone explained 30 % of the variance (marginal R^2^ = 0.297), while the combined fixed and random effects explained 65 % of the variance (conditional R^2^ = 0.648). Adding age group as a fixed effect to the analysis showed no main effect of age group on social information use (*M* adults = 0.39, *M* adolescents = 0.36, *b* = −0.03, β = −0.06, *p* = 0.131), which is in contrast with earlier studies finding more social information use in adolescence (Andrews et al., 2021, Somerville, 2013). Notably, when our participants performed a similar task, albeit without any certainty manipulations, approximately half an hour later, adolescents relied significantly more on social information than adults (*M* adults = 0.31, *M* adolescents = 0.38, *b* = 0.07, β = 0.17, *p* = 0.003, Figure S1). This task difference emerged despite a positive correlation in average social information use across the two tasks (*r* = 0.23, *p* = 0.010). This may suggest that adolescents typically use more social information, but the presence of certainty and confidence cues in our task may have reduced this tendency. Crucially, age group did modulate the extent to which own certainty affected social information use (*b* = 0.09, β = 0.08, *p* = 0.001), such that adolescents were less sensitive to own certainty than adults (Fig. 2B,C). Age group did not significantly modulate the effect of peer confidence on social information use (*b* = −0.02, β = −0.03, *p* = 0.219), nor was there a significant three-way interaction between own certainty, peer confidence and age group (*b* = 0.02, β = 0.02, *p* = 0.102). To further explore the interaction between age group and own certainty, we used estimated marginal means to examine age-related differences at each level of own certainty. This revealed that the effect was driven by the uncertain condition, such that adolescents used less social information than adults in the uncertain condition (*t*(130) = 2.50, *p* = 0.014) but not in the certain condition (*t*(130) = -0.48, *p* = 0.635). Although adolescents were overall less sensitive to own certainty than adults, they still showed a significant effect of own certainty on social information use (adolescents: *t*(130) = 3.82, *p* < 0.001; adults: *t*(130) = 8.65, *p* < 0.001). An exploratory analysis on the participants’ mean confidence ratings, which they reported after each trial during the practice round (1 = low, 2 = medium, 3 = high confidence), revealed a similar effect. The difference between reported confidence in the uncertain versus the certain condition was smaller for adolescents compared to adults (*b* = −0.473, *p* < 0.001; Fig. 2D). Estimated marginal means revealed that this was mainly driven by adolescents feeling more confident than adults in the uncertain (adolescents: 1.99; adults: 1.60; *t*(258) = -4.30, *p* < 0.001) but not the certain condition (adolescents: 2.20; adults: 2.28; *t*(258) = 0.89, *p* = 0.377). To ensure group age differences in social information use were not driven by group differences in performance, we examined performance, indexed as deviance of participant’s first estimate from the true percentage of blue mushrooms as a function of group. This did not reveal a significant difference in performance between age groups (*b* = 0.04, *p* = 0.888; Fig. 2E), nor did performance depend on own certainty (*b* = −0.08, *p* = 0.715) or the interaction between group and own certainty (*b* = −0.05, *p* = 0.919). We next tested whether adolescents differ from adults in their ability to align confidence with performance. However, there was no significant group difference in the correlation between deviance and confidence ratings (*R*^*2*^ adolescents = 0.119, *R*^*2*^ adults = 0.118, *t* = 0.03, *p* = 0.975, Fig. 2F). Together, this suggests that age-related differences in the effect of own certainty on social information use are unlikely to be explained by differences in performance or in the mapping of performance to subjective confidence.

The effects reported above are based on a linear mixed-effects model that assumes homoscedastic residuals, or equivalent noise across age groups and conditions. As a control, we also tested whether allowing for age-group-specific residual variances would change the results. The heteroscedastic model showed improved model fit, but the fixed effects remained consistent, suggesting that the findings are robust to differences between age groups in noisy responding (Supplementary Section *Modeling Heteroscedasticity*).

### 3.3 Computational mechanisms of social information use

**Fig. 3:**
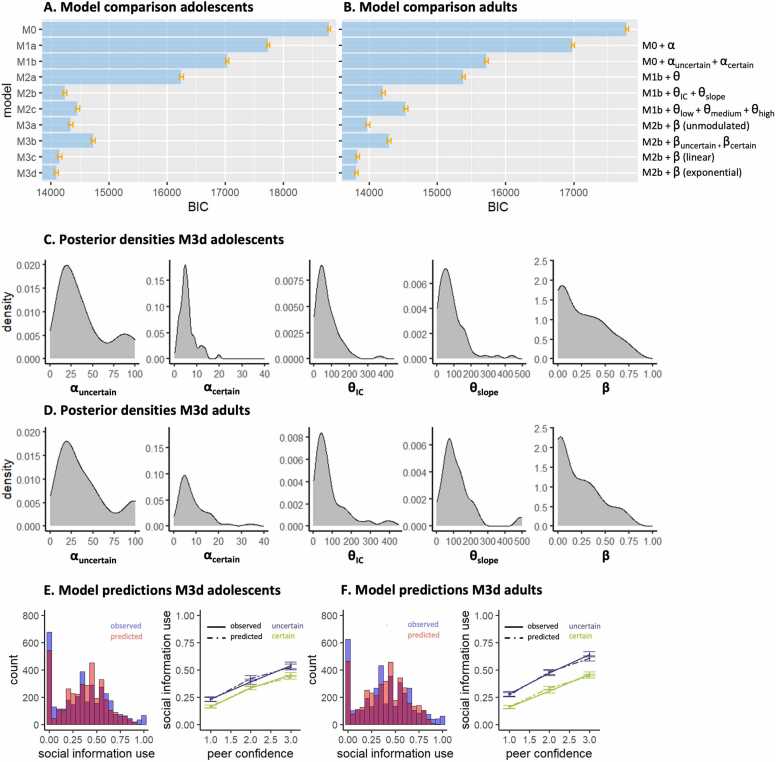
**Model evidence and parameter inference**. Model evidence, relative to the best fitting model M3d, for adolescents **(A)** and adults **(B)**. Stepwise addition of a parameter that modulates the weight of the personal information (α) a parameter that modulates the weight of the peer information (θ) and a stay bias (β) improves model fit, quantified by BIC. Lower BIC indicates better model fit. Posterior densities of the best fitting model M3d for adolescents **(C)** and adults **(D)**. Posterior predictive model simulations of the best fitting model M3d for adolescents **(E)** and adults **(F)**. The left panel of each figure shows predicted (red) and observed (blue) social information use across all trials, whereas the right panel of each figure shows the predicted (dotted lines) and observed (solid lines) for each own certainty and peer confidence level. Error bars represent the standard error around the mean.

**Fig. 4:**
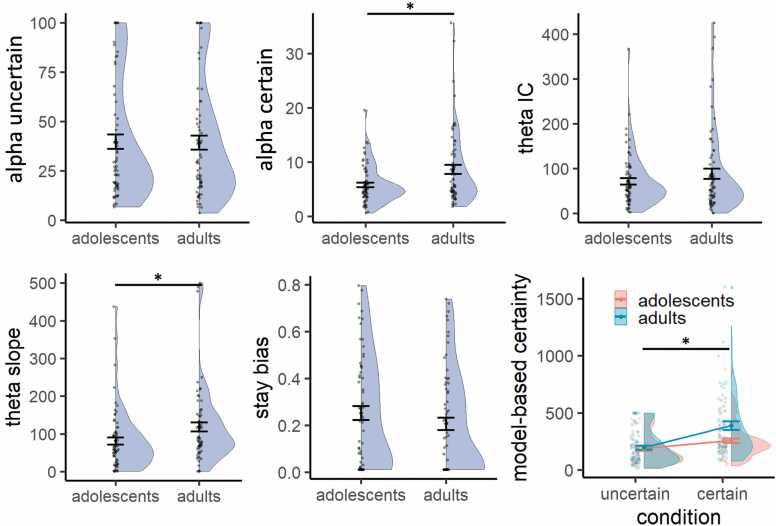
**Parameter estimates**. Parameter estimates for adolescents and adults. Mann-Whitney U tests revealed significant group-level differences in the modulation of personal information in the certain condition ($\alpha_{\mathrm{certain}}$) and in the sensitivity to peer confidence (θ_slope_). Bottom right figure depicts model based certainty for both own certainty conditions, calculated by multiplying participants’ $\alpha_{5}$ and $\alpha_{45}$ with the actual number of mushrooms. The difference in model based certainty between the uncertain and certain condition is significantly smaller for adolescents compared to adults. Error bars represent standard error around the mean. Individual points represent participants.

We used a computational modeling approach to quantify mechanisms hypothesized to govern social information use. Model comparison showed that, for both age groups, the model that best captured the data was M3d (Fig. 3A,B; Table 1), which included a dual α to modulate the impact of own certainty on the weight of the personal information at E1, a $\theta_{\mathrm{IC}}$ and $\theta_{\mathrm{slope}}$ that together modulated the weight of the peer estimate, linearly dependent on peer confidence, and a stay bias β to capture participants tendency to stay with their initial estimate, which became exponentially smaller as peer confidence increased:

Fig. 3C and D illustrate the parameter estimates and model predictions per age group. The dual α parameter allowed for a differential conversion of the actual number of mushrooms in each condition into a subjective count, reflecting differential translation into model-based certainty. Participants amplified their model-based certainty more in the uncertain condition (median $\alpha_{\mathrm{uncertain}}$ = 29.4) compared to the certain condition (median $\alpha_{\mathrm{certain}}$ = 5.3). When we multiply participants’ $\alpha_{\mathrm{uncertain}}$ and $\alpha_{\mathrm{certain}}$ with the actual number of mushrooms, we see that their model-based certainty was significantly lower in the uncertain (median = 146.8) versus the certain condition (median = 239.3; *V* = 1178, *p* < 0.001; Fig. 4 bottom right panel), such that participants behaved *as if* they were almost twice as certain in the certain versus the uncertain condition, even though the actual ratio between 45 and 5 is 9. Participants thus discounted the condition difference, mainly driven by a relatively exaggerated model-based certainty in the uncertain condition.

Further improvement of the model fit due to the addition of the θ parameters provides clear evidence of participants behaving *as if* the peer had seen more mushrooms for higher reported peer confidence. As per Eq. 9, a median $\theta_{\mathrm{IC}}$ of 54.8 and $\theta_{\mathrm{slope}}$ of 79.0 resulted in participants behaving *as if* the peer had seen 54.8 mushrooms for low confidence, 133.9 mushrooms for median confidence and 212.9 mushrooms for high confidence. Consequently, higher peer confidence led to a stronger weighting of the peer’s estimate in the participant’s second and final estimate.

Finally, better model performance after adding a stay bias that depended on peer confidence, but not on own certainty, shows that peer confidence is the driver of this decision heuristic. This indicates that even though both own certainty and peer confidence affected *the extent* to which participants used social information, changes of mind were more likely when peer confidence was high, but was not different for low versus high own certainty.

### 3.4 Model recovery and parameter recovery

The model recovery results show that data generated by one specific model generally also resulted in the best model fit for that same model, indicating successful model recovery (Figure S2). Importantly, simulations were not falsely fit best by our winning model M3d, suggesting that M3d does not overfit data generated by other models. However, there was some overlap in model fits between M3c and M3d, which is unsurprising given their strong similarity (a stay bias that depends linearly versus exponentially on peer confidence level.

Our parameter recovery procedure on M3d revealed high Pearson’s correlations between the ‘true’ and fitted parameter estimates (all *r* ≥ 0.60, all *p* < 0.001; Table S2) suggesting that the model was adequately able to recover the parameters (see Table S3 for correlations between the different parameters).

### 3.5 Age differences in computational processes

To evaluate differences in cognitive processes between adolescents and adults, we subsequently tested each parameter for statistical differences using a Mann-Whitney *U* test (Fig. 4). This revealed no significant difference in *α* in the uncertain condition (median $\alpha_{\mathrm{uncertain}}$ adolescents = 28.4; median $\alpha_{5}$ adults = 29.5; *W* = 2095, *p* = 0.714, *p*_*corrected*_ = 0.769), but a significant lower α in the certain condition for adolescents compared to adults (median $\alpha_{\mathrm{certain}}$ adolescents = 4.9; median $\alpha_{45}$ adults = 5.9; *W* = 1629, *p* = 0.013, *p*_*corrected*_ = 0.032). This indicates that compared to adults, adolescents experienced a similar model-based certainty in the uncertain condition (adolescents: 28.4 * 5 = 142; adults: 29.5 * 5 = 148), but a lower model-based certainty in the certain condition (adolescents: 4.9 * 45 = 221; adults: 5.9 * 45 = 266). Consequently, this led to lower model-based difference in certainty between the conditions for adolescents compared to adults (*W* = 1482, *p* = 0.002; Fig. 4 bottom right). This is opposite to the pattern we found in the model-free analysis, where we saw that the group difference in sensitivity to own certainty was mainly driven by higher reported confidence and lower social information use in the *uncertain* condition for adolescents compared to adults.

Regarding peer confidence, there was no significant group difference in $\theta_{\mathrm{IC}}$ (median adolescents = 54.2; median adults = 55.3; *W* = 2111, *p* = 0.769, *p*_*corrected*_ = 0.769), indicating that adolescents and adults did not differ in how they interpreted low peer confidence. However, there was a significant group difference in $\theta_{\mathrm{slope}}$ (median adolescents = 62.0; median adults = 89.2; *W* = 1604.5, *p* = 0.009, *p*_*corrected*_ = 0.032). This indicates that with each increase in peer confidence level, adolescents behaved *as if* the peer had seen 62 more mushrooms, whereas adults behaved *as if* the peer had seen 89 more mushrooms. Thus, even though both groups allocated more weight to the peer estimate when peer confidence increased, compared to adults, adolescents allocated less weight to peer estimates when peer confidence was medium and high. This age-related difference was not present in the model-free results, highlighting the added value of the computational analyses in capturing subtle differences in how adolescents discount peer estimates more than adults, primarily at higher confidence levels.

There was no significant group difference in the β value for the stay bias (median adolescents = 0.22; median adults: 0.15; *W* = 2527.5, *p* = 0.103, *p*_*corrected*_ = 0.172), suggesting that adolescents and adults were equally likely to change their minds after seeing the peer estimate.

### 3.6 Neural activity in response to model-based own certainty and peer confidence across age groups

**Fig. 5:**
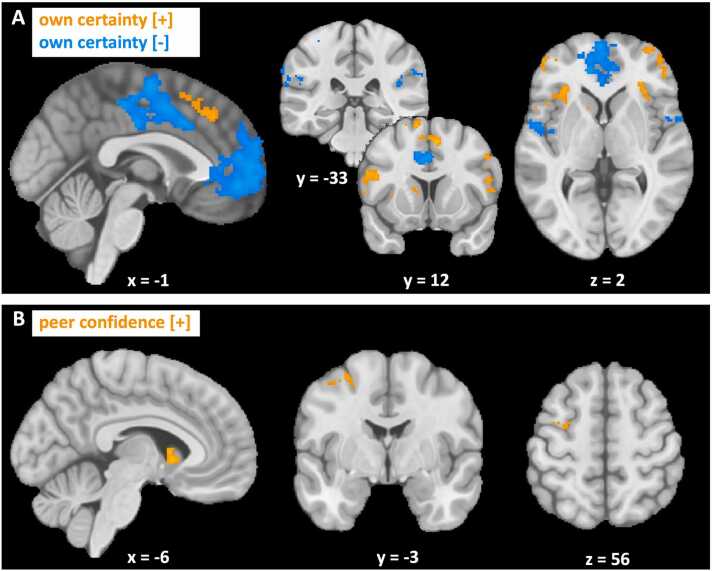
**Neural activity in response to model-based own certainty and peer confidence across age groups**. Clusters showing BOLD signal that significantly increased (orange) or decreased (blue) in response to **(A)** own certainty or **(B)** peer confidence. Cluster-level corrected, FWE, p < 0.05. Coordinates correspond to MNI space.

First, we assessed neural activity at the time of the first estimate as a function of model-based own certainty (Fig. 5A, Table S4). There was a large network of regions that showed a positive correlation with certainty (certain > uncertain), including the dorsomedial PFC (dmPFC) and the bilateral dlPFC, anterior insula and left caudate nucleus. A different network showed a negative correlation with certainty (uncertain > certain), including the supplementary motor area, the mPFC, the bilateral Rolandic operculum, adjacent to the posterior insula, and areas within the bilateral TPJ. Results from our supplemental GLM that included model-free regressors of own certainty and peer confidence rendered results comparable to the individually-modeled regressors, validating the neural underpinnings of our computational model (Table S4; Figure S3C).

Next, we looked at neural activity at the time when the social information was presented as a function of model-based peer confidence (Fig. 5B, Table S5). Neural activity in the left precentral gyrus and caudate nucleus correlated positively with peer confidence, whereas a small cluster in the right middle frontal gyrus correlated negatively with peer confidence. Again, we found similar clusters for model-free peer confidence (Table S5; Figure S3D).

### 3.7 Age differences in neural activity in response to model-based own certainty and peer confidence

**Fig. 6:**
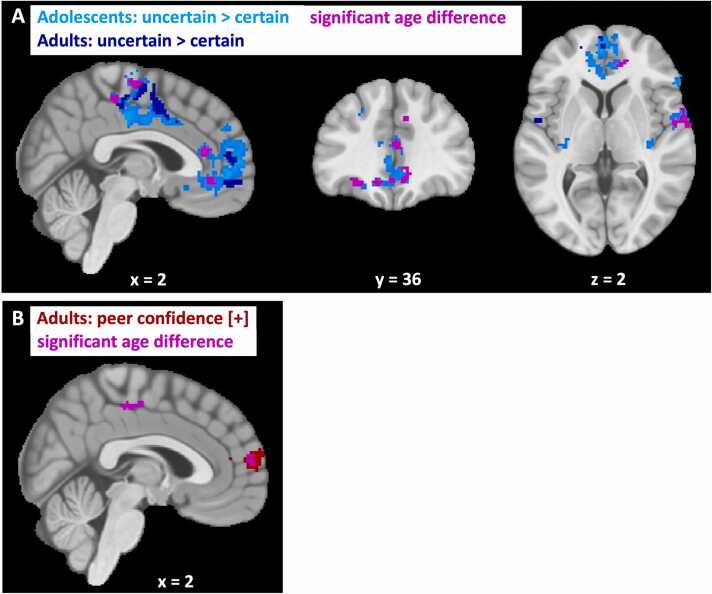
**Age differences in neural activity in response to model-based own certainty and peer confidence across age groups**. Clusters showing significant differences between age groups (pink) in response to model-based **(A)** own certainty and **(B)** peer confidence. Cluster-level corrected, FWE, p < 0.05. Coordinates correspond to MNI space.

When comparing neural activity in response to model-based own certainty in adolescents versus adults at the time of the first estimate, we found that adolescents showed a stronger negative correlation in the anterior cingulate cortex and mPFC, the right Rolandic operculum and supplemental motor area (Fig. 6A; Table S6, also for model-free results). Indeed, post hoc analyses revealed that when analyzing the groups separately, we found a strong negative correlation of own certainty in these regions for adolescents and less so or not significantly for adults. This suggests that adolescents were more sensitive to own certainty than adults at the neural level, which is in contrast with the behavioral and computational results, which showed lower sensitivity to own certainty in adolescents. We therefore speculated that this higher neural sensitivity for adolescents at the time of the first estimate E1 might well have disappeared at the time of the social information, when personal and social information are being combined, such that at the time of social information, adults would show greater sensitivity to own certainty than adolescents. To investigate this, we ran the same model-based and model-free fMRI analyses again, but now modeled own certainty at the time of the social information rather than at E1. This revealed no differences in neural activity between adolescents and adults for either ways of modeling own certainty. Thus, whereas there was an age difference in sensitivity to own certainty, this was no longer significant at the time of integrating personal and social information.

When comparing neural activity in response to model-based peer confidence in adolescents versus adults at the time of social information, we observed significant differences in neural activity within the anterior and ventral parts of the mPFC and the mid-cingulate cortex for adults compared to adolescents (Fig. 6B; Table S7, also for model-free results). Analyzing the groups separately revealed a significant positive correlation with peer confidence in the mPFC for adults but not for adolescents. Conversely, no significant correlation was observed for either of the separate groups in the mid-cingulate cortex.

### 3.8 Correlation between neural and behavioral sensitivity to peer confidence

**Fig. 7:**
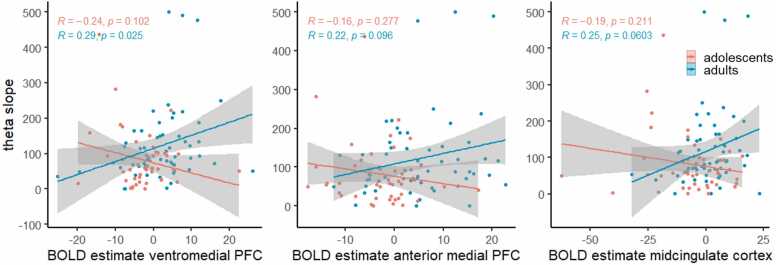
**Correlations between model-free BOLD contrast estimates and model-derived sensitivity to peer confidence (θ**_**slope**_**)**. Correlation for adolescents (red) and adults (blue) in three clusters showing age-related differences in neural sensitivity to peer confidence: ventromedial prefrontal cortex, anterior medial prefrontal cortex and midcingulate cortex. Scatterplots show individual data points with linear regression lines per group.

We wanted to further validate the link between neural activity and individual differences in computational parameters. We limited our brain–behavior correlation analysis to peer confidence, as age differences in sensitivity to own certainty did not persist at the time of information integration and were opposite in direction across neural and behavioral levels. To this aim, we extracted mean subject-level BOLD contrast estimates from 3 mm-radius spherical ROIs centered on the peak voxel of each of the three group-level clusters showing age-related differences in sensitivity to peer confidence. We identified these clusters using the model-based GLM as described above. However, as this GLM already includes the subject-specific parametric values from individual model fits, the resulting beta estimates are incomparable across participants. We therefore extracted BOLD contrast estimates from the model-free GLM in which peer confidence was modeled as a parametric modulator defined identically across participants (i.e. condition-based). We then correlated the BOLD contrast estimates with each individual’s computationally derived θ_slope_, which reflects behavioral sensitivity to peer confidence. As expected, this revealed age-group difference in brain-behavior correlations (ventromedial PFC: Fisher’s *z* = 2.72, *p* = 0.007; anterior medial PFC: Fisher’s *z* = 1.92, *p* = 0.055; midcingulate cortex: Fisher’s *z* = 2.18, *p* = 0.029; Fig. 7), with adolescents showing no significant correlation between BOLD signal and θ_slope_ (ventromedial PFC: *r* = -0.24, *p* = 0.102; anterior medial PFC: *r* = -0.16, *p* = 0.277; midcingulate cortex: *r* = -0.19, *p* = 0.211), but adults showing a (marginally) significant positive correlation (ventromedial PFC: *r* = 0.29, *p* = 0.025; anterior medial PFC: *r* = 0.22, *p* = 0.096; midcingulate cortex: *r* = 0.25, *p* = 0.060). These findings suggest that the link between neural and computational sensitivity to peer confidence strengthens with age.

## 4 Discussion

In this study, we examined the neurocognitive processes of social information use, and compared whether these differed between adolescents and adults. Overall, we found that participants across age groups relied more on their own estimates than on social information, but flexibly adjusted their behavior depending on their own certainty and the confidence expressed by a peer. Adolescents and adults showed broadly similar decision-making processes, though a combination of model-free and model-based analyses revealed that adolescents were less sensitive to their own certainty and peer confidence. The neural data showed overlapping brain responses across age groups in regions previously linked to uncertainty and social cognition, including the dlPFC, mPFC, insula, caudate nucleus and TPJ. However, adolescents showed heightened neural responses to their own certainty in anterior cingulate and medial prefrontal regions, though this difference was no longer present at the stage of information integration. In contrast, adults exhibited stronger neural responses to peer confidence in the vmPFC. Below, we first discuss the behavioral patterns of social information use, before turning to the neural data.

Roughly similar to previous studies (Gradassi et al., 2022a, Molleman et al., 2019, Yaniv, 2004), we find that people rely more on their personal information than on the social information, a phenomenon known as egocentric discounting. Independent of age, our data revealed that participants used more social information when they were uncertain themselves and when the peer reported to be more confident. Moreover, own certainty and peer confidence interacted, such that social information use was more strongly impacted by peer confidence when people were uncertain, to a degree where social information use well exceeded 0.5. This interactive effect is in line with recent findings (Gradassi et al., 2022a), and shows that the egocentric bias can be overcome in situations where social information is most likely to be beneficial. Our model-based results were in line with our model-free results. For both groups, behavior was best explained by a model that combined Bayesian information processing based on precision weighing of the personal and social information with a bias to stay with the first estimate. This means that participants assigned a higher weight to their personal information in the certain condition, and they assigned a higher weight to the social information when the peer reported to be more confident. A stay bias that was independent of this Bayesian process allowed for participants’ tendency to stick with their initial estimate, in line with earlier work finding that social information use can best be described by a combination of Bayesian decision-making and simple heuristics (Molleman et al., 2020). This suggests that social information, especially low quality information, is often ignored, even though it could in principle still improve participants’ estimates when sufficiently taking into account the level of precision.

Adolescents and adults did not differ in terms of overall social information use. This is in contrast with the intuitive and empirically-informed prediction that adolescents would use more information. Previous experimental studies did find that adolescents were more influenced by social information than adults (Andrews et al., 2021, Somerville, 2013). Interestingly, when approximately half an hour after the mushroom task the same individuals performed a similar perceptual decision-making task (Berlin Estimate Adjustment Task; (Molleman et al., 2019)), albeit it without any certainty or confidence manipulations, we found that adolescents used more social information than adults. This suggests that adolescents are more inclined to use social information when reliability cues are absent. In contrast, adults may be more hesitant to rely on social information unless there is a clear indication of its reliability or a strong need for additional information. Alternatively, we speculate that the complexity of the current task (e.g. longer trial duration, multiple screens to display personal and social information, multiple levels of own certainty and peer confidence) could have led the younger group of participants to rely more on one particular set of information, conceivably their personal information, rather than integrating all information available to them to the extent they would have done during a less demanding task. Thus, while our findings did not replicate a group-level difference in overall social information use, they do not necessarily contradict the notion of adolescence as a period of heightened social sensitivity. Instead, our data suggest that adolescents' social information use might be more context-dependent: in the absence of explicit reliability cues, they may be more inclined than adults to rely on social information, or conversely, under increased task complexity, they may default more strongly than adults to their own information.

**Fig. 8:**
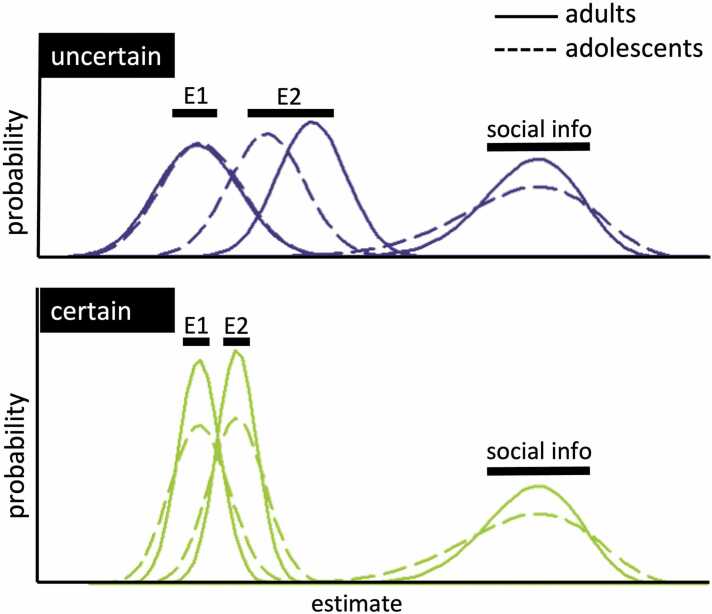
**Model-derived subjective representations of Bayesian information integration for adolescents and adults**. Top: In the uncertain condition, adolescents and adults showed similar subjective mushroom counts for their own estimate (prior; E1), but adolescents perceived the peer's reported mushroom count as lower than adults at high peer confidence levels. This results in a flatter (less precise) probability distribution for peer information among adolescents, leading to smaller updates in their second estimates (posterior; E2) and reduced social information use compared to adults. Bottom: In the certain condition, adolescents showed lower subjective mushroom counts for their own estimate compared to adults, indicating lower internal certainty (wider prior). Peer information remained perceived as less precise than in adults. While adolescents' second estimates remained more uncertain than those of adults, this resulted in similarly weighted updates between groups.

Compared to adults, adolescents showed a smaller effect of the own certainty condition on social information, mainly driven by adolescents’ lower social information use in the uncertain condition compared to adults. The effect of peer confidence did not differ across age groups. Both adolescents and adults used more social information when the peer reported to be more confident, similar to people’s response to indicators of intelligence and social status (Gradassi et al., 2022b, Helms et al., 2014, da Silva Pinho et al., 2021). Our computational model provided a deeper understanding of the cognitive process by breaking it down into distinct components, offering more nuanced insights compared to the model-free analysis. While adolescents exhibited a smaller difference between the certain and uncertain conditions, consistent with the model-free findings, the model revealed that this difference was driven by adolescents behaving as if they were less certain than adults in the certain condition. Additionally, the model highlighted that adolescents were less sensitive to peer confidence levels while showing no differences in stay bias compared to adults. The apparent contrast between the model-free and model-based findings can be understood by examining how the Bayesian model integrates subjective uncertainty. According to the model-based results, in the *uncertain* condition, adolescents and adults did not differ in how many mushrooms they subjectively perceived in their own estimate. However, adolescents perceived the peer's estimate as less precise than adults did, especially when the peer expressed high confidence (Fig. 8, top). In Bayesian terms, this means that adolescents assigned a wider (i.e., less precise) distribution to the peer information. As a result, they gave less weight to the peer's input when updating their estimate, which matches the lower social information use observed in the model-free data. In contrast, in the *certain* condition, adolescents subjectively perceived fewer mushrooms than adults did in their own estimate. This implies they assigned less precision (i.e., a wider prior) to their own information. At the same time, they continued to perceive the peer's estimate as less precise than adults did (Fig. 8, bottom). This led to a situation where both sources, personal and social, were seen as relatively imprecise by adolescents, resulting in an update that was similar in size to that of adults. This is consistent with the lack of a group difference in social information use in the certain condition as seen in the model-free results. The key difference lies in how certain participants were about their final (second) estimate: in the certain condition, adolescents showed greater uncertainty in their second estimate than adults, as indicated by the wider posterior distribution (E2). This suggests that while the amount of adjustment may appear similar in model-free terms, adolescents were still internally less confident in their final judgments, a difference that is only captured by the model-based analysis. Together, these findings highlight how computational modeling can reveal latent cognitive differences that remain hidden in behavioral outcomes.

Consistent with our results, previous studies have indeed shown that adolescents often display behavior indicative of greater uncertainty compared to adults in learning tasks (Javadi et al., 2014, Jepma et al., 2020). These observations may be related to still maturing metacognitive skills of adolescents (Moses-Payne et al., 2021, Weil et al., 2013). While our data suggest that adolescents and adults do not differ in how they translate their own performance into subjective confidence reports, adolescents may nonetheless be less accurate in appraising the uncertainty in the environment. This may account for their reduced ability to perform precision-weighting and to accurately differentiate between varying levels of certainty, as well as peer confidence statements (Reiter et al., 2021). An alternative explanation is that adolescents are less ambiguity averse than adults (Tymula et al., 2012, Van Den Bos and Hertwig, 2017), and may therefore feel less need to resolve ambiguity by incorporating peer information when uncertain about their own estimate. This could result in lower social information use specifically in the uncertain condition. However, this explanation appears to be less consistent with the observed computational results: if adolescents were simply less ambiguity averse, we would expect them to assign relatively high precision to their own estimates and rely less on the peer. Instead, the computational model shows that adolescents behaved as if they had lower subjective certainty about both their own and the peer’s information, particularly in the certain condition. This pattern suggests not a reduced need to resolve ambiguity, but rather a difference in how adolescents weigh uncertainty.

Together, our behavioral findings suggest that while adolescents and adults both rely on social information influenced by their own certainty and peer confidence, adolescents show a smaller distinction between different levels of own certainty and peer confidence, possibly due to ongoing neural and metacognitive development. Despite these differences, the core processes of social information use appear similar across age groups, combining Bayesian decision-making with heuristic biases.

To gain more insights into the neural mechanisms underlying sensitivity to own certainty and peer confidence, we compared the neural activity of adolescents adults using fMRI. Firstly, this revealed a large overlap in neural processing between the two groups. We found that neural activity correlated positively with own certainty mainly in the dorsal parts of the PFC, the anterior insula and the caudate nucleus. A negative correlation, on the other hand, was mainly present in the more anterior and ventral parts of the mPFC, the posterior parts of the insula and the Rolandic operculum and areas within the TPJ. Many of these areas have indeed been implicated in the cognitive processing of uncertainty-related information. For example, the anterior insula and dlPFC have been linked to processing risk and uncertainty (Badre et al., 2012, Feldmanhall et al., 2019, Singer et al., 2009), while vmPFC activation is thought to reflect confidence signals (Gherman and Philiastides, 2018, Lebreton et al., 2015, Rouault et al., 2023), though these signals can sometimes manifest in opposite directions. In addition to processing confidence signals, activation in the vmPFC in response to uncertainty, along with activation in the TPJ, may suggest that participants are proactively preparing to process the upcoming peer information when their own evidence base is limited, aligning with the TPJ’s role in social processing and attention to peers (Jin et al., 2023, Sul et al., 2017, Van Overwalle, 2011). Involvement of the caudate nucleus in processing both own certainty and peer confidence might be due to its role in value signaling (Delgado et al., 2004; Grahn et al., 2008), with more information in the certain condition and peer information associated with higher confidence levels being more valuable. Importantly, a similar set of regions whose activity correlated with own certainty and peer confidence as set by the task manipulations also correlated with the model-based estimates of own certainty and peer confidence. This suggests that the neural patterns align with our Bayesian model of social information use, even though our model may be too simplistic to capture the entire decision-making process.

When comparing age differences in neural activity, we observed larger neural responses to differences in own certainty in adolescents compared with adults, including in the Rolandic operculum, the anterior regions of the mPFC and cingulate cortex and the supplementary motor area. This is surprising, as we found stronger sensitivity at the behavioral level for adults. However, the increased neural responsivity in adolescents was no longer present at the time of the peer information, suggesting that this strong initial response in adolescents was not utilized when integrating personal and social information. This might point at poorer metacognition in adolescence compared to adulthood (Moses-Payne et al., 2021, Weil et al., 2013), leading to an aberrant translation of initially available neurocognitive information to decisional output in adolescence, which might in turn stem from, subsequently affecting the search or use of additional information (Schulz et al., 2023).

Although we were initially quite agnostic about the cognitive differences in sensitivity to peer confidence between adults and adolescents, we found that adults showed a stronger response than adolescents, particularly in the vmPFC, which aligns with our model-based results. As mentioned earlier, this region is heavily implicated in both confidence processing and social cognition. Lower responsiveness in the vmPFC might therefore mean that adolescents possess a lower ability to distinguish between varying levels of peer confidence. This could result from decreased recruitment of Theory-of-Mind processes to modulate attention to peer information and aligns with prior research showing that learning for others but not for the self, as reflected by prediction error coding in the vmPFC, showed an age related increase during adolescence (Westhoff et al., 2021). However, other regions related to social cognition and especially Theory-of-Mind, including the TPJ, do not exhibit differential neural activity between adolescents and adults. Another possible explanation comes from findings that the vmPFC plays a crucial role in the integration of information (Campbell-Meiklejohn et al., 2017, Kahnt et al., 2011, Park et al., 2020). Notably, brain–behavior correlations showed that BOLD signal in the vmPFC and anterior mPFC correlated with individual differences in model-based sensitivity to peer confidence only in adults and not in adolescents. Thus, our findings might indicate that this region is functionally less developed or active in adolescents compared to adults when it comes to integrating personal and social information.

The strength of the current design, combined with our computational model, lies in its ability to disentangle the various components of social information use under uncertainty. However, it is not without its limitations. One limitation is the order of the presentation of personal and peer information. It is hard to distinguish the distinct neurocognitive contributions of one's own certainty, peer confidence, and the integration of the two at the second time point, as participants may begin integrating the information as soon as they see the peer's input. Future studies could vary whether participants receive their personal or the peer's information first, enabling researchers to assess how each type of information is processed independently before integration. Additionally, such a design could reveal whether the sequence of information presentation affects the overall decision-making process. Another limitation of our study is how information was presented across conditions. In the uncertain condition, participants were given a small amount of clear and easily memorable information, while in the certain condition, they received a larger volume of more reliable information, presented rapidly. This introduces a potential confound between inferential certainty (where the uncertain condition was less certain) and perceptual certainty (where participants might feel more confident about what they saw in the uncertain condition, despite it being harder to interpret). This distinction could have influenced the neural signals of certainty or uncertainty, potentially explaining why some of our findings contrast with the directions reported in other neuroimaging studies. Future research should consider adjusting how information is presented, such that inferential certainty is manipulated while perceptual certainty is aligned across conditions.

An interesting avenue for future research is to more accurately pinpoint belief precision during Bayesian social information use. Even though our results suggest that adolescents exhibit lower sensitivity to certainty and confidence due to a more limited integration of precision signals, future studies could test this in more detail at the neural level by employing a neuroimaging design in which the timings are optimized to model neural activity in response to each successive piece of personal or social information (Skowron et al., 2024). This could be combined with a larger and more varied range of ages within the adolescent sample to assess any continuous effects of age to evaluate how and when neurocognitive mechanisms of social information use and precision weighing develop during adolescence. Ideally, a longitudinal design would be employed to preclude any cohort effects, such that participants are tested multiple times from early adolescence through late adolescence or early adulthood.

While we did not directly assess how participants interpreted the peer confidence ratings, the peer had sampled from different fields with potentially different numbers of mushrooms, which suggests that peer confidence reflected informational resources. However, we acknowledge that we cannot be sure whether participants interpreted higher peer confidence as better informational resources, general expertise, or even trait characteristics. This makes it difficult to determine whether social information use in our task was driven primarily by informational influence (aiming to improve one’s own accuracy) or by normative influence (aiming to align with a confident peer). Importantly, these motives may differ across development. While adults may be more selective in whose information they trust based on perceived expertise (Hofmans and van den Bos, 2022), adolescents may be more sensitive to normative social influence and place greater weight on social information when it comes from peers who are similar to them in age or identity, or with whom they share a close relationship. A related question is how individuals gradually learn about the confidence or trustworthiness of a peer, rather than being provided with an explicit rating as in the current study. This process of learning and integrating social information is likely to be influenced by various factors, including an individual's prior experiences (e.g. interactions with trustworthy or untrustworthy peers) (Olsen et al., 2019). Future studies could explore how prior experiences shape the interpretation of peer information.

## 5 Conclusion

In sum, our findings contribute to the growing understanding of how adolescents and adults process and integrate social information. While both age groups exhibit similar core mechanisms, such as Bayesian decision-making combined with heuristic biases, notable differences emerge in how own certainty and peer confidence influence their behavior and neural responses. Adolescents appear to show reduced sensitivity to variations in their own certainty and peer confidence, possibly due to ongoing neural and metacognitive development, which may hinder their ability to effectively integrate precision signals with the relevant information.

## CRediT authorship contribution statement

**Lieke Hofmans:** Writing – review & editing, Writing – original draft, Visualization, Software, Project administration, Methodology, Investigation, Formal analysis, Data curation, Conceptualization. **Wouter van den Bos:** Writing – review & editing, Supervision, Methodology, Funding acquisition, Conceptualization.

## Declaration of Competing Interest

The authors declare that they have no known competing financial interests or personal relationships that could have appeared to influence the work reported in this paper.

## Footnotes

1 Trials in which social information use (s) fell outside the 0–1 range were excluded, as such values are not captured by the modeling framework, which assumes s reflects a weighted average of own and peer estimates. However, rerunning the model-free analysis with those trials included yielded equivalent results.
