# Supplementary material for: Developmental differences in social information use under uncertainty: A neurocomputational approach
Source: Dev Cogn Neurosci. 2025 Aug 7;75:101604. doi: 10.1016/j.dcn.2025.101604 (PMC12365119; doi:10.1016/j.dcn.2025.101604)
Supplement: Table S1 — Supplementary material [file mmc1.docx]

| **Table S1. Exclusions (participants *N* and trials) based on behavioral data.** | | | | | |
| --- | --- | --- | --- | --- | --- |
|  |  |  |  |  |  |
| **ADULTS** |  |  |  |  |  |
|  | ***N*** | **Trials** | **% of total trials** |  |  |
| Complete datasets; passed qualitative inspection; non-filler trials | 70 | 4200 | 100.0 |  |  |
| **Exclusions** |  |  |  |  |  |
| Missed trials | 0 | 25 | 0.6 |  |  |
| Trials without suitable social information | 0 | 2 | 0.1 |  |  |
| Outlier estimates (E1 or E2) | 0 | 89 | 2.1 |  |  |
| s < 0 | 0 | 43 | 1.0 |  |  |
| s > 1 | 0 | 8 | 0.2 |  |  |
| s = 0 on more than 70% of trials | 2 | 114 | 2.7 | **-** |  |
| **Net total** | 68 | 3919 | 93.3 |  |  |
|  |  |  |  |  |  |
| **ADOLESCENTS** |  |  |  |  |  |
|  | ***N*** | **Trials** | **% of total trials** |  |  |
| Complete datasets; passed qualitative inspection; non-filler trials | 70 | 4200 | 100.0 |  |  |
| **Exclusions** |  |  |  |  |  |
| Missed trials | 0 | 20 | 0.5 |  |  |
| Trials without suitable social information | 0 | 8 | 0.2 |  |  |
| Outlier estimates (E1 or E2) | 0 | 137 | 3.3 |  |  |
| s < 0 | 0 | 87 | 2.1 |  |  |
| s > 1 | 0 | 18 | 0.4 |  |  |
| s = 0 on more than 70% of trials | 6 | 327 | 7.8 | **-** |  |
| **Net total** | 64 | 3610 | 86.0 |  |  |

**
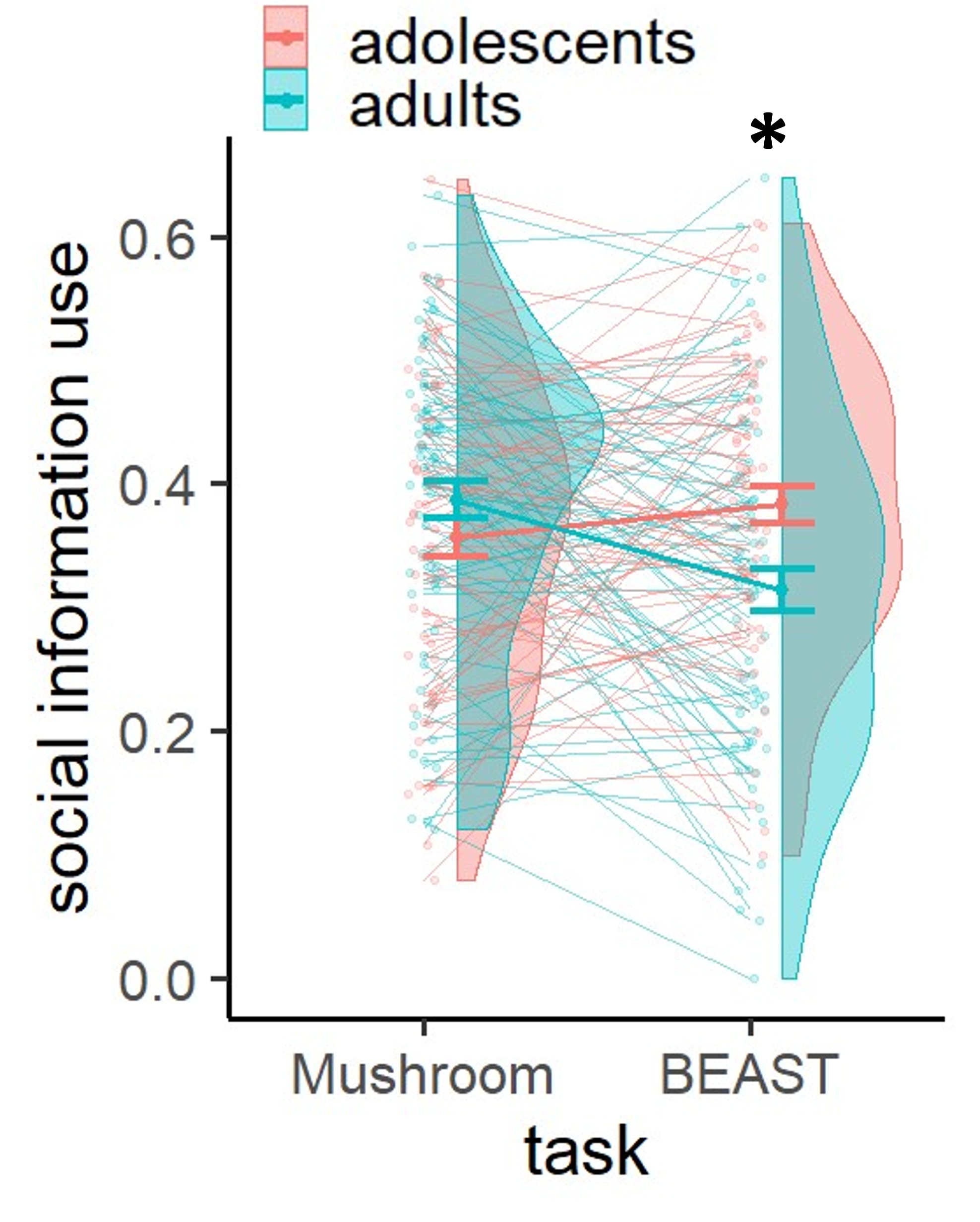
**

**Figure S1. Social information use across tasks.** Social information use by the same group of adult and adolescent participants across two tasks. The Mushroom task, described in the main text of this manuscript, was performed during fMRI scanning. The Berlin Estimate Adjustment Task (Molleman et al., 2019) was performed approximately 30 minutes later, outside the scanner. This latter task involved estimating the number of animals on the screen, after which participants were shown the estimate made by an unknown peer and they could revise their initial estimate. Average social information use, computed as described in the Methods section in the main text, in the Mushroom task was 0.36 for adolescents and 0.39 for adults (*t*(129.1) = -1.37, *p* = 0.172). Average social information use in the BEAST was 0.38 for adolescents and 0.31 for adults (*t*(126.05) = 3.00, *p* = 0.003). Error bars represent the standard error around the mean.

**Modeling Heteroscedasticity**

To evaluate whether noisy decision-making differed by age group, we computed the variance of social information use for each participant in each condition (age group × own certainty × peer confidence). We then log-transformed the variances and ran a linear model including all main effects and interactions:

$$\log\left( variance \right)\sim own certainty \left( ref=uncertain \right)*peer confidence*group \left( ref= adults \right)$$

This revealed a significant main effect of age group (*b* = 0.34, *p* = 0.030), such that the variance in social information use was higher for adolescents compared to adults. There were no significant interactions between age and the other variables (all *p >* 0.390).

We then fitted an additional model using the lme function from the *nlme* package to assess whether differences in residual variability influenced the effects on social information use. This heteroscedastic model included the same fixed and random effects structure as our main lmer model but allowed the residual variance to differ by age group. Using a likelihood ratio test, we compared this heteroscedastic model to the homoscedastic model used in the main analyses. This revealed that model fit indeed improved when allowing for unequal variances across age groups (χ²(1) = 57.6, *p* < 0.001). However, the estimated fixed effects did not meaningfully change (age group: *b* = -0.03, *p* = 0.131; age group × own certainty: *b* = 0.09, *p* = 0.001; age group × peer confidence: *b* = -0.02, *p* = 0.218), suggesting our main conclusions are robust to heteroscedasticity or more noisy decision-making by adolescents compared to adults.


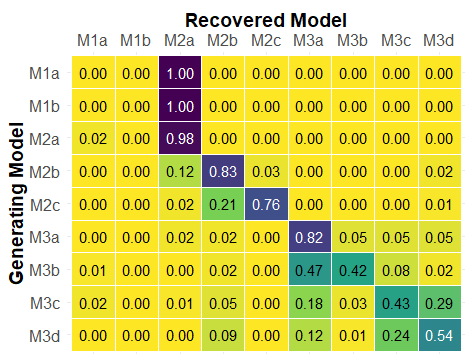


**Figure S2. Model recovery results.** Each model was used to generate simulated data for 132 participants, after which each of these models was fit to these 9 simulated datasets. Model fits were compared using the Bayesian Information Criterion (BIC), and a confusion matrix was constructed to quantify model recoverability. Each entry in this matrix represents the proportion of subjects for whom a given model provided the best fit (lowest BIC) to data generated by each model.

| **Table S2. Parameter recovery: Pearson’s correlations between simulated and fitted parameter values across both age groups.** | | | | | | |
| --- | --- | --- | --- | --- | --- | --- |
|  |  |  |  |  |  |  |
| **M1a** |  |  |  | **M3a** |  |  |
| parameter | *r* | *p* |  | parameter | *r* | *p* |
| α | 0.99 | < 0.001 |  | α_uncertain_ | 0.68 | < 0.001 |
|  |  |  |  | α_certain_ | 0.70 | < 0.001 |
| **M1b** |  |  |  | θ_IC_ | 0.67 | < 0.001 |
| parameter | *r* | *p* |  | θ_slope_ | 0.56 | < 0.001 |
| α_uncertain_ | 0.95 | < 0.001 |  | β | 0.89 | < 0.001 |
| α_certain_ | 0.84 | < 0.001 |  |  |  |  |
|  |  |  |  | **M3b** |  |  |
| **M2a** |  |  |  | parameter | *r* | *p* |
| parameter | *r* | *p* |  | α_uncertain_ | 0.79 | < 0.001 |
| α_uncertain_ | 0.41 | < 0.001 |  | α_certain_ | 0.76 | < 0.001 |
| α_certain_ | 0.47 | < 0.001 |  | θ_IC_ | 0.75 | < 0.001 |
| θ | 0.53 | < 0.001 |  | θ_slope_ | 0.70 | < 0.001 |
|  |  |  |  | β_uncertain_ | 0.92 | < 0.001 |
| **M2b** |  |  |  | β_certain_ | 0.76 | < 0.001 |
| parameter | *r* | *p* |  |  |  |  |
| α_uncertain_ | 0.80 | < 0.001 |  | **M3c** |  |  |
| α_certain_ | 0.63 | < 0.001 |  | parameter | *r* | *p* |
| θ_IC_ | 0.80 | < 0.001 |  | α_uncertain_ | 0.69 | < 0.001 |
| θ_slope_ | 0.77 | < 0.001 |  | α_certain_ | 0.61 | < 0.001 |
|  |  |  |  | θ_IC_ | 0.71 | < 0.001 |
| **M2c** |  |  |  | θ_slope_ | 0.64 | < 0.001 |
| parameter | *r* | *p* |  | β | 0.92 | < 0.001 |
| α_uncertain_ | 0.77 | < 0.001 |  |  |  |  |
| α_certain_ | 0.75 | < 0.001 |  | **M3d** |  |  |
| θ_low_ | 0.75 | < 0.001 |  | parameter | *r* | *p* |
| θ_medium_ | 0.78 | < 0.001 |  | α_uncertain_ | 0.60 | < 0.001 |
| θ_high_ | 0.81 | < 0.001 |  | α_certain_ | 0.61 | < 0.001 |
|  |  |  |  | θ_IC_ | 0.64 | < 0.001 |
|  |  |  |  | θ_slope_ | 0.61 | < 0.001 |
|  |  |  |  | β | 0.95 | < 0.001 |

| **Table S3. Pearson’s correlations between model parameters (M3d).** | | | | | |
| --- | --- | --- | --- | --- | --- |
|  |  | $\alpha_{uncertain}$ | $\alpha_{certain}$ | $\theta_{IC}$ | $\theta_{slope}$ |
|  | $\alpha_{uncertain}$ |  |  |  |  |
|  | $\alpha_{certain}$ | 0.50 (*p* < .001) |  |  |  |
|  | $\theta_{IC}$ | 0.48 (*p* < .001) | 0.51 (*p* < .001) |  |  |
|  | $\theta_{slope}$ | 0.35 (*p* < .001) | 0.65 (*p* < .001) | 0.50 (*p* < .001) |  |
|  | $\beta$ | 0.07 (*p* = .453) | -0.15 (*p* = .088) | -0.35 (*p* < .001) | -0.31 (*p* < .001) |
| Note: High correlations between parameter could potentially lead to erroneously fitted values. However, our parameter recovery procedure indicated that these values could be adequately recovered (Table S2). | | | | | |

**Age as a continuous predictor**

In our primary analyses, we treated age as a categorical variable (adolescents vs adults). However, to assess the robustness of our findings, we also conducted supplementary analyses using age as a continuous predictor for behavioral and model-based outcomes.

Regarding the model-free analyses, we ran the same model as for the main analyses, but now with age as a mean-centered continuous predictor.

$$s \sim own certainty \left( ref=uncertain \right)*peer confidence*age+\left( 1+own certainty*peer confidence \right| participant)$$

The results mirror those of the analysis with age as a group-wise predictor. Again, there was no significant effect of age on social information use (*b* < 0.001, *p* = 0.910). Age modulated the extent to which own certainty affected social information use (*b* = -.006, *p* = 0.036), such that younger participants were less sensitive to own certainty than older participants. There was no interaction with peer confidence level (*b* < 0.001, *p* = 0.570), nor was there a three-way interaction between own certainty, peer confidence and age (*b* = -0.001, *p* = 0.384).

To test the relationship between age and the fitted parameter values that resulted from our computational model, we ran a linear model in which age predicted each of the five parameter values. Similar to the group-wise analysis, this revealed a significant relationship between age and α in the certain condition (*b* = 0.25, *p_uncorrected_* = 0.008), such that younger participants behaved *as if* they were less certain than older participants in the certain condition, which in turn led to a smaller difference between the uncertain and certain condition for younger participants. Age was not significantly related to the other parameter values (α_uncertain_: *b* = 0.33, *p* = 0.500; θ_IC_: *b* = 0.83, *p* = 0.543; θ_slope_: *b* = 2.43, *p* = 0.123; β: *b* < 0.001 *p* = 0.848).

Thus, except for the non-significant effect of age on the parameter values for θ_slope_, the results broadly mirrored the group-based findings, suggesting that our main conclusions are not an artifact of the age categorization.

| **Table S4. Brain regions associated with own certainty during the first estimate (E1).** | | | | | | | | |
| --- | --- | --- | --- | --- | --- | --- | --- | --- |
|  |  |  |  |  |  | **Peak coordinates** | | |
|  | **Region** | **Hemisphere** | **Max *z*-value** | **N voxels** | ***p*-value** | **x** | **y** | **z** |
| **Positive correlation model-based own certainty** | | | | |  |  |  |  |
|  | Inferior/middle frontal gyrus | Right | 6.43 | 1733 | < 0.001 | 60 | 16 | 34 |
|  | Middle frontal gyrus | Left | 5.43 | 653 | < 0.001 | -49 | 42 | 20 |
|  | Supplemental motor area / medial frontal gyrus | Right | 5.45 | 390 | < 0.001 | 8 | 12 | 52 |
|  | Inferior frontal gyrus | Left | 5.39 | 217 | < 0.001 | -59 | 16 | 24 |
|  | Anterior insula | Right | 5.76 | 210 | < 0.001 | 30 | 24 | 10 |
|  | Anterior insula | Left | 5.47 | 145 | < 0.001 | -31 | 30 | -3 |
|  | Caudate nucleus | Left | 4.54 | 80 | < 0.001 | -17 | -1 | 20 |
|  | Supplemental motor area | Left | 4.58 | 48 | 0.003 | -11 | 12 | 66 |
| **Negative correlation model-based own certainty** | | | | |  |  |  |  |
|  | mPFC | Left | 5.94 | 2201 | < 0.001 | -1 | 50 | -7 |
|  | Supplemental motor area / mid-cingulate cortex | Left | 5.72 | 1326 | < 0.001 | -1 | -9 | 62 |
|  | Precentral gyrus | Left | 5.79 | 1054 | < 0.001 | -29 | -23 | 78 |
|  | Postcentral gyrus | Left | 4.85 | 199 | < 0.001 | -63 | -21 | 48 |
|  | Rolandic operculum | Left | 4.46 | 187 | < 0.001 | -53 | -3 | 4 |
|  | Precentral gyrus | Right | 5.14 | 174 | < 0.001 | 42 | -13 | 54 |
|  | Superior frontal gyrus | Left | 4.74 | 133 | < 0.001 | -23 | 44 | 42 |
|  | Supramarginal gyrus (TPJ) | Right | 4.81 | 106 | < 0.001 | 54 | -45 | 30 |
|  | Rolandic operculum | Right | 5.30 | 88 | < 0.001 | 48 | 2 | 12 |
|  | Supramarginal gyrus (TPJ) | Left | 4.17 | 68 | < 0.001 | -71 | -31 | 34 |
|  | Superior frontal gyrus | Right | 4.15 | 63 | < 0.001 | 12 | 50 | 48 |
|  | Precentral gyrus | Left | 4.77 | 41 | 0.009 | -63 | 4 | 34 |
|  | Supramarginal gyrus (TPJ) | Right | 4.45 | 40 | 0.010 | 56 | -37 | 30 |
|  | Posterior insula | Left | 4.17 | 36 | 0.019 | -39 | 2 | -13 |
|  | Superior temporal gyrus (TPJ) | Right | 4.59 | 35 | 0.022 | 44 | -35 | 22 |
|  | Rolandic operculum | Right | 4.57 | 31 | 0.041 | 38 | -19 | 18 |
| **Positive correlation model-free own certainty** | | |  |  |  |  |  |  |
|  | Inferior/middle frontal gyrus | Right | 7.86 | 4493 | < 0.001 | 46 | 42 | 28 |
|  | Anterior insula / inferior frontal gyrus | Left | 6.87 | 1544 | < 0.001 | -33 | 28 | -1 |
|  | Supplemental motor area / medial frontal gyrus | Left | 6.81 | 1333 | < 0.001 | -5 | 24 | 46 |
|  | Caudate nucleus | Left | 6.29 | 263 | < 0.001 | -15 | 14 | 12 |
|  | Caudate nucleus | Right | 6.12 | 193 | < 0.001 | 14 | 18 | 6 |
|  | Middle frontal gyrus | Left | 5.18 | 191 | < 0.001 | -33 | 56 | 18 |
| **Negative correlation model-free own certainty** | | |  |  |  |  |  |  |
|  | Precentral gyrus / supplemental motor area/mid-cingulate cortex | Left | 9.00 | 4423 | < 0.001 | -41 | -25 | 58 |
|  | mPFC | Right | 6.68 | 2784 | < 0.001 | 2 | 56 | 8 |
|  | Precentral gyrus | Right | 6.13 | 374 | < 0.001 | 34 | -13 | 68 |
|  | Rolandic operculum | Left | 5.56 | 344 | < 0.001 | -51 | -1 | 6 |
|  | Rolandic operculum | Right | 5.66 | 236 | < 0.001 | 48 | 2 | 10 |
|  | Superior temporal gyrus (TPJ) | Right | 6.52 | 201 | < 0.001 | 48 | -35 | 22 |
|  | Nucleus accumbens | Right | 5.92 | 142 | < 0.001 | 6 | 14 | -5 |
|  | Nucleus accumbens | Left | 5.47 | 123 | < 0.001 | -5 | 12 | -3 |
|  | Angular gyrus (TPJ) | Right | 5.00 | 93 | < 0.001 | 66 | -51 | 28 |
|  | Precuneus | Left | 5.22 | 87 | < 0.001 | -1 | -47 | 64 |
|  | Putamen | Left | 4.67 | 79 | 0.001 | -25 | -9 | -5 |
|  | Posterior insula | Right | 5.16 | 69 | 0.003 | 36 | -25 | 10 |
|  | Supramarginal gyrus (TPJ) | Left | 4.58 | 51 | 0.016 | -63 | -31 | 26 |
|  | Postcentral gyrus | Left | 5.68 | 51 | 0.016 | -61 | -17 | 22 |

| **Table S5. Brain regions associated with peer confidence when social information is displayed.** | | | | | | | | |
| --- | --- | --- | --- | --- | --- | --- | --- | --- |
|  |  |  |  |  |  | **Peak coordinates** | | |
|  | **Region** | **Hemisphere** | **Max *z*-value** | **N voxels** | ***p*-value** | **x** | **y** | **z** |
| **Positive correlation model-based peer confidence** | | | |  |  |  |  |  |
|  | Caudate nucleus | Left | 4.98 | 51 | < 0.001 | -7 | 10 | -1 |
|  | Precentral gyrus | Left | 4.06 | 33 | 0.015 | -29 | -5 | 58 |
| **Negative correlation model-based peer confidence** | | | |  |  |  |  |  |
|  | Middle frontal gyrus | Right | 4.27 | 33 | 0.015 | 34 | 60 | 24 |
| **Positive correlation model-free peer confidence** | | |  |  |  |  |  |  |
|  | Precentral gyrus | Left | 4.71 | 166 | < 0.001 | -53 | 6 | 38 |
|  | Precentral gyrus | Left | 5.10 | 133 | < 0.001 | -31 | -3 | 60 |
|  | Precentral gyrus | Right | 4.43 | 96 | < 0.001 | 32 | -7 | 54 |
|  | Precentral gyrus | Left | 4.33 | 78 | < 0.001 | -49 | -17 | 52 |
|  | Caudate nucleus | Right | 4.81 | 64 | 0.002 | 8 | 6 | 4 |
|  | Caudate nucleus | Left | 4.59 | 44 | 0.020 | -7 | 10 | -1 |
| **Negative correlation model-free peer confidence** | | |  |  |  |  |  |  |
|  | Postcentral gyrus | Right | 4.87 | 103 | < 0.001 | 26 | -39 | 68 |
|  | Middle frontal gyrus | Right | 4.08 | 41 | 0.029 | 36 | 62 | 16 |
|  | Middle frontal gyrus | Right | 4.34 | 38 | 0.042 | 38 | 32 | 50 |


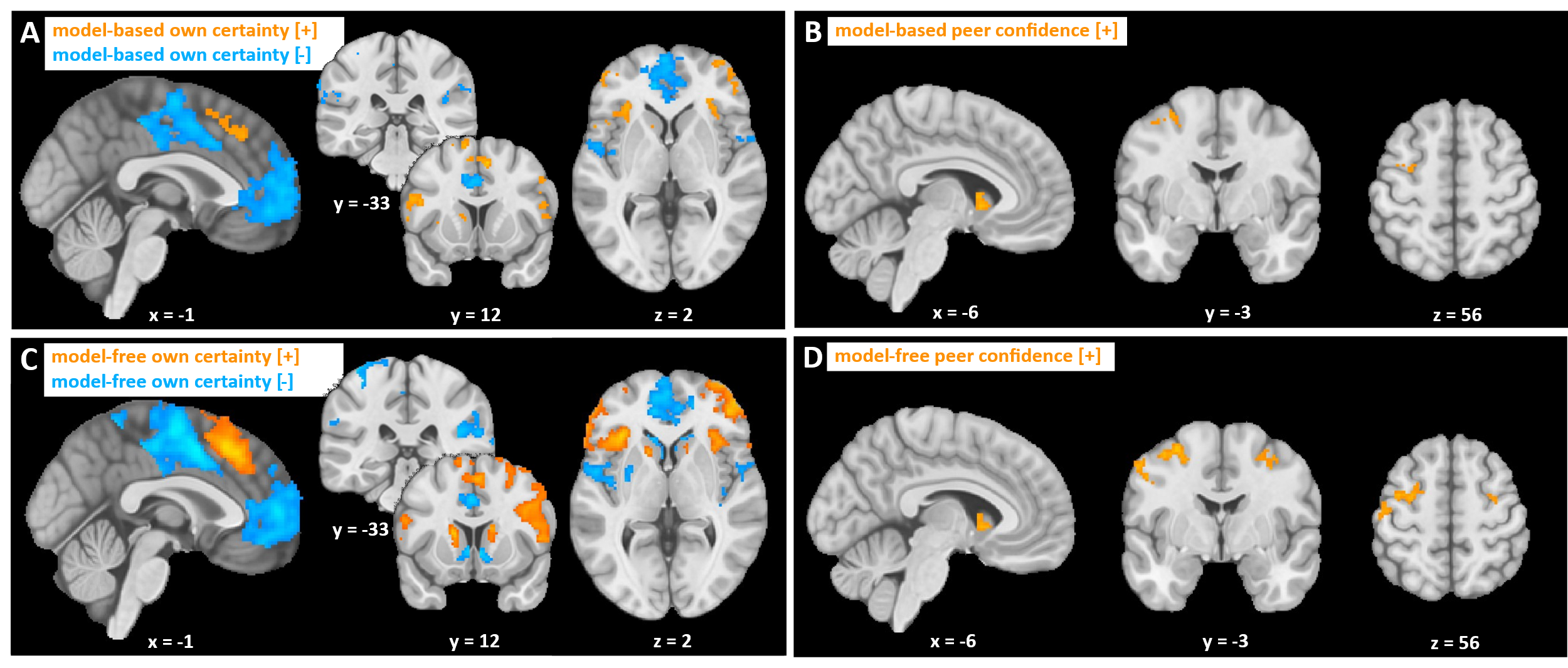
**Figure S3. Neural activity in response to own certainty and peer confidence across age groups.** Upper panels depict clusters showing BOLD signal that significantly increased (orange) or decreased (blue) in response to *model-based* **(A)** own certainty or **(B)** peer confidence. Model-based own certainty and peer confidence are based on the computationally derived parameter values for α and θ, respectively. Lower panels depict clusters showing BOLD signal that significantly increased (orange) or decreased (blue) in response to *model-free* **(C)** own certainty or **(D)** peer confidence. Model-free own certainty (uncertainty, certain) and peer confidence (low, medium, high) are based on task conditions. Cluster-level corrected, FWE, p < 0.05. Coordinates correspond to MNI space.

| **Table S6. Brain regions associated with significant age differences in response to own certainty during the first estimate (E1).** | | | | | | | | |
| --- | --- | --- | --- | --- | --- | --- | --- | --- |
|  |  |  |  |  |  | **Peak coordinates** | | |
|  | **Region** | **Hemisphere** | **Max *z*-value** | **N voxels** | ***p*-value** | **x** | **y** | **z** |
| **Model-based own certainty** | | | |  |  |  |  |  |
| **(adults > adolescents)** | |  |  |  |  |  |  |  |
|  | Anterior cingulate cortex | Right | 4.46 | 189 | < 0.001 | 14 | 44 | 6 |
|  | Rolandic operculum | Right | 4.17 | 94 | < 0.001 | 60 | 4 | -1 |
|  | mPFC | Right | 4.59 | 74 | < 0.001 | 10 | 50 | 26 |
|  | Mid-cingulate cortex | Right | 4.20 | 55 | 0.001 | 2 | -29 | 54 |
|  | Precentral gyrus | Right | 4.30 | 53 | 0.002 | 24 | -27 | 70 |
|  | Supplemental motor area | Right | 3.81 | 43 | 0.007 | 4 | -13 | 64 |
|  | Inferior frontal gyrus | Left | 4.57 | 40 | 0.010 | -27 | 34 | -13 |
|  | Anterior cingulate cortex | Right | 4.22 | 30 | 0.048 | 2 | 34 | 14 |
| **Model-based own certainty** | | | |  |  |  |  |  |
| **(adolescents > adults)** | |  |  |  |  |  |  |  |
|  | Precentral gyrus | Left | 3.85 | 35 | 0.022 | -49 | 4 | 42 |
| **Model-free own certainty** | | | |  |  |  |  |  |
| **(adults > adolescents)** | |  |  |  |  |  |  |  |
|  | Postcentral gyrus | Right | 4.47 | 171 | < 0.001 | 20 | -25 | 62 |
|  | Postcentral gyrus | Left | 4.94 | 149 | < 0.001 | -25 | -29 | 66 |
|  | Rolandic operculum | Right | 4.50 | 75 | 0.001 | 60 | -1 | 2 |
|  | Supplemental motor area | Right | 3.91 | 42 | 0.041 | 2 | -17 | 64 |
| **Model-free own certainty** | | | |  |  |  |  |  |
| **(adolescents > adults)** | |  |  |  |  |  |  |  |
|  | - |  |  |  |  |  |  |  |
| Note: This table shows the interactive effect between age group and own certainty. A more positive effect for adults versus adolescents (adults > adolescents) might indicate either a stronger positive or a weaker negative effect of own certainty for adults versus adolescents. Vice versa, a more positive effect for adolescents versus adults (adolescents > adults) might indicate either a stronger positive or a weaker negative effect of own certainty for adolescents versus adults.  Note: no age differences were present anymore at the time of social information. | | | | | | | | |

| **Table S7. Brain regions associated with significant age differences in response to peer confidence when social information is displayed.** | | | | | | | | |
| --- | --- | --- | --- | --- | --- | --- | --- | --- |
|  |  |  |  |  |  | **Peak coordinates** | | |
|  | **Region** | **Hemisphere** | **Max *z*-value** | **N voxels** | ***p*-value** | **x** | **y** | **z** |
| **Model-based peer confidence** | | | |  |  |  |  |  |
| **(adults > adolescents)** | |  |  |  |  |  |  |  |
|  | Mid-cingulate cortex | Right | 4.05 | 45 | 0.002 | 2 | -25 | 50 |
|  | Anterior mPFC | Right | 3.94 | 35 | 0.011 | 6 | 60 | 10 |
|  | Ventromedial PFC | Right | 4.41 | 27 | 0.044 | 14 | 44 | -7 |
| **Model-based peer confidence** | | | |  |  |  |  |  |
| **(adolescents > adults)** | |  |  |  |  |  |  |  |
|  | - |  |  |  |  |  |  |  |
| **Model-free peer confidence** | | |  |  |  |  |  |  |
| **(adults > adolescents)** | |  |  |  |  |  |  |  |
|  | Anterior mPFC | Right | 4.64 | 108 | < 0.001 | 2 | 60 | 10 |
|  | Ventromedial PFC | Right | 4.16 | 78 | < 0.001 | 10 | 48 | 4 |
| **Model-free peer confidence** | | |  |  |  |  |  |  |
| **(adolescents > adults)** | |  |  |  |  |  |  |  |
|  | Inferior frontal gyrus | Left | 4.03 | 53 | 0.007 | -45 | 44 | 8 |
| Note: This table shows the interactive effect between age group and peer confidence. A more positive effect for adults versus adolescents (adults > adolescents) might indicate either a stronger positive or a weaker negative effect of peer confidence for adults versus adolescents. Vice versa, a more positive effect for adolescents versus adults (adolescents > adults) might indicate either a stronger positive or a weaker negative effect of peer confidence for adolescents versus adults. | | | | | | | | |

| **Table S8. Brain regions associated with significant age differences in response to own certainty during the first estimate (E1) – whole brain analysis** | | | | | | | | |
| --- | --- | --- | --- | --- | --- | --- | --- | --- |
|  |  |  |  |  |  | **Peak coordinates** | | |
|  | **Region** | **Hemisphere** | **Max *z*-value** | **N voxels** | ***p*-value** | **x** | **y** | **z** |
| **Model-based own certainty** | | | |  |  |  |  |  |
| **(adults > adolescents)** | |  |  |  |  |  |  |  |
|  | Anterior cingulate cortex | Right | 4.46 | 189 | < 0.001 | 14 | 44 | 6 |
|  | Superior temporal sulcus | Right | 4.65 | 121 | < 0.001 | 64 | -1 | -9 |
|  | Superior temporal sulcus | Left | 4.46 | 87 | < 0.001 | -53 | -15 | -9 |
|  | mPFC | Right | 4.59 | 74 | < 0.001 | 10 | 50 | 26 |
|  | Mid-cingulate cortex | Right | 4.20 | 58 | 0.001 | 2 | -29 | 54 |
|  | Mid-cingulate cortex | Right | 4.54 | 55 | 0.002 | 2 | -21 | 38 |
|  | Precentral gyrus | Right | 4.30 | 53 | 0.002 | 24 | -27 | 70 |
|  | Supplemental motor area | Right | 3.81 | 43 | 0.008 | 4 | -13 | 64 |
|  | Inferior frontal gyrus | Left | 4.57 | 40 | 0.012 | -27 | 34 | -13 |
|  | Putamen | Right | 4.22 | 36 | 0.023 | 24 | 6 | -11 |
|  | Superior temporal gyrus | Right | 4.20 | 33 | 0.036 | 66 | -17 | 10 |
| **Model-based own certainty** | | | |  |  |  |  |  |
| **(adolescents > adults)** | |  |  |  |  |  |  |  |
|  | Precentral gyrus | Left | 3.85 | 35 | 0.026 | -49 | 4 | 40 |
| Note: This table shows the interactive effect between age group and own certainty. A more positive effect for adults versus adolescents (adults > adolescents) might indicate either a stronger positive or a weaker negative effect of own certainty for adults versus adolescents. Vice versa, a more positive effect for adolescents versus adults (adolescents > adults) might indicate either a stronger positive or a weaker negative effect of own certainty for adolescents versus adults.  Note: no age differences were present anymore at the time of social information. | | | | | | | | |

| **Table S9. Brain regions associated with significant age differences in response to peer confidence when social information is displayed – whole brain analysis** | | | | | | | | | |
| --- | --- | --- | --- | --- | --- | --- | --- | --- | --- |
|  |  |  |  |  |  | **Peak coordinates** | | | |
|  | **Region** | **Hemisphere** | **Max *z*-value** | **N voxels** | ***p*-value** | **x** | | **y** | **z** |
| **Model-based peer confidence** | | | |  |  |  | |  |  |
| **(adults > adolescents)** | |  |  |  |  | |  |  |  |
|  | Mid-cingulate cortex | Right | 4.05 | 47 | 0.002 | 2 | | -25 | 50 |
|  | Anterior mPFC | Right | 3.94 | 35 | 0.013 | 6 | | 60 | 10 |
| **Model-based peer confidence** | | | |  |  |  | |  |  |
| **(adolescents > adults)** | |  |  |  |  | |  |  |  |
|  | - |  |  |  |  |  | |  |  |
| Note: This table shows the interactive effect between age group and peer confidence. A more positive effect for adults versus adolescents (adults > adolescents) might indicate either a stronger positive or a weaker negative effect of peer confidence for adults versus adolescents. Vice versa, a more positive effect for adolescents versus adults (adolescents > adults) might indicate either a stronger positive or a weaker negative effect of peer confidence for adolescents versus adults. | | | | | | | | | |
